# Supplementary material for: A district-level ensemble model to enhance dengue prediction and control for the Mekong Delta Region of Vietnam
Source: PLoS Negl Trop Dis. 2025 Sep 29;19(9):e0013571. doi: 10.1371/journal.pntd.0013571 (PMC12507206; doi:10.1371/journal.pntd.0013571)
Supplement: S1 — (DOCX) [file pntd.0013571.s001.docx]

**S1: Additional Figures and Tables**

**
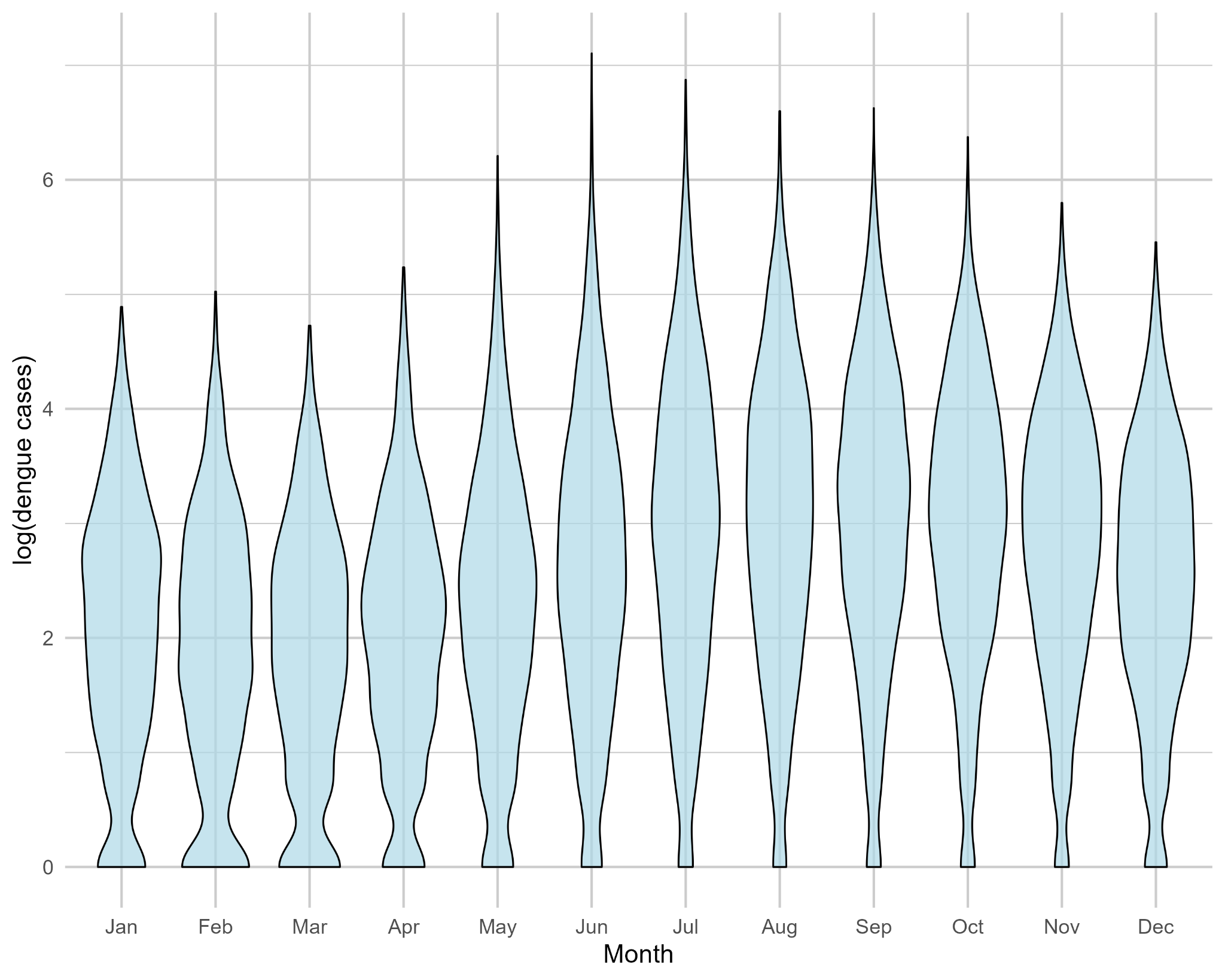
**

Fig A: Monthly dengue incidence (log scale) from 2004 to 2022. The shaded area reflects the density of cases, with wider sections indicating higher density. Case variability was highest from June to October, aligning with the rainy season and increased transmission.

Table A : Socio-economic dataset (data source: General Statistics Office of Vietnam)

| Name | Unit | Frequency | Level of data |
| --- | --- | --- | --- |
| Population density | Km^2^ | Yearly | District |
| Population data | pop | Yearly | District |
| In-migration rate | % | Yearly | Province |
| Poverty rate | % | Yearly | Province |
| Hygienic water access | % | Yearly | Province |
| Hygienic toilet access | % | Yearly | Province |
| Monthly average income | VND^a^ | Yearly | Province |
| Monthly average income per capita | VND^a^ | Yearly | Province |
| Total passenger by province each year | Million-person time | Yearly | Province |
| Urbanization rate | % | Yearly | Province |
| Special land use (includes land used by the government offices, public services, construction facilities, security and national defence land, land for non-agricultural production and business, and public land) | m^2^ | Yearly | Province |

### ^a^ Vietnamese dong

Table B : Preventive measures/entomologic indices dataset (data source: Vietnam National Surveillance System)

| **Name** | **Unit** | **Frequency** | **Level of data** |
| --- | --- | --- | --- |
| Larvae index (BI) | No unit | Monthly | District |
| CI^b^ larvae | % | Monthly | District |
| HI^c^ larvae | % | Monthly | District |
| Mosquito index (DI^d^) | % | Monthly | District |
| HI^c^ mosquitoes | % | Monthly | District |
| Breeding site elimination campaigns | Interventions (N) | Monthly | District |
| Active spraying | Intervention (N) | Monthly | District |
| Large scale spraying for epidemic response | Intervention (N) | Monthly | District |
| Communication and/or training | Intervention (N) | Monthly | District |
| Number of outbreaks detected | Outbreaks | Monthly | District |
| Number of outbreak responses | Outbreaks | Monthly | District |

^a^ Breteau index; ^b^ Container index; ^c^ House index; ^d^ Density index

**
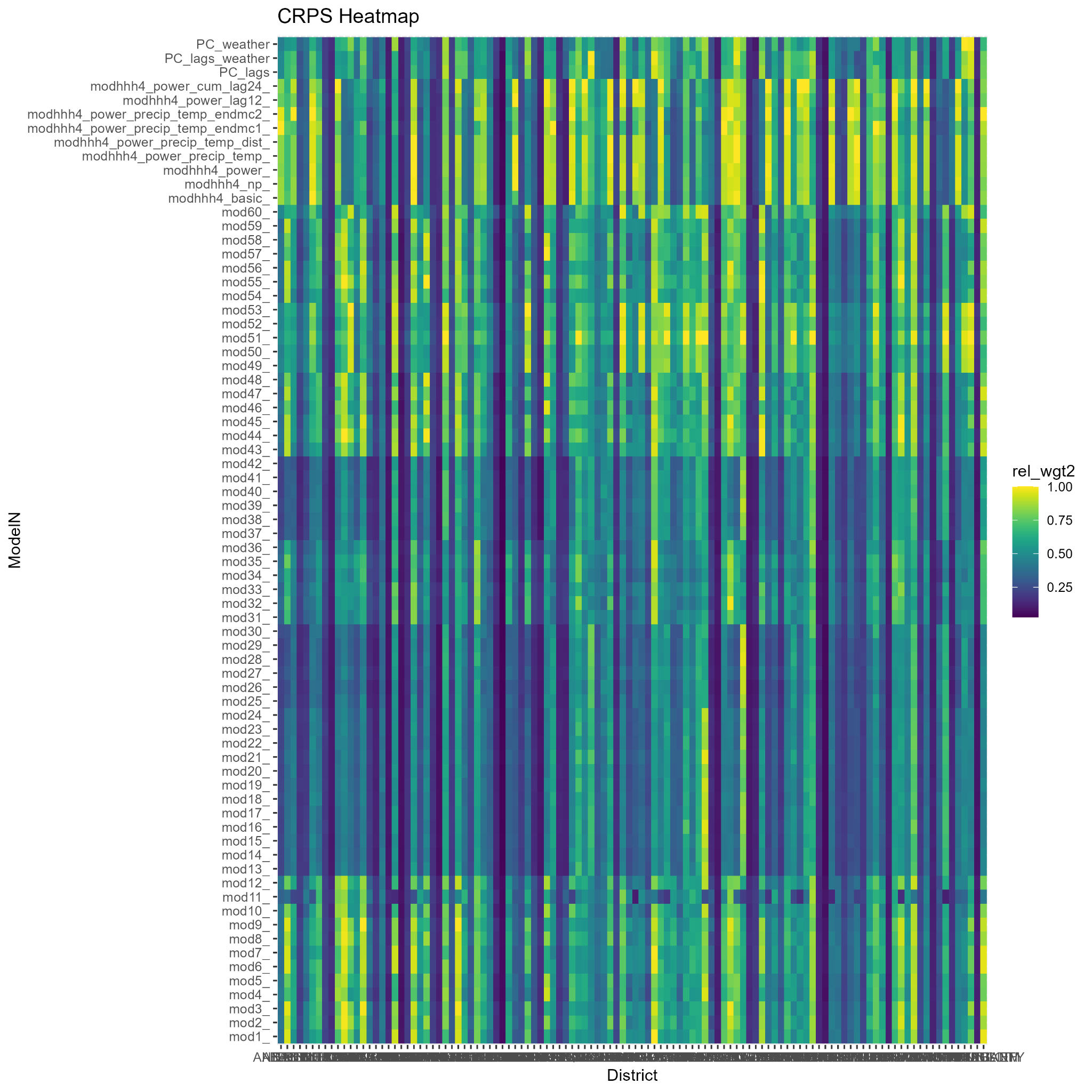
**

Fig B: Heat map of CRPS relative values, with the x-axis representing districts and the y-axis showing the tested models; each cell shows a model’s CRPS divided by the mean CRPS for that district.


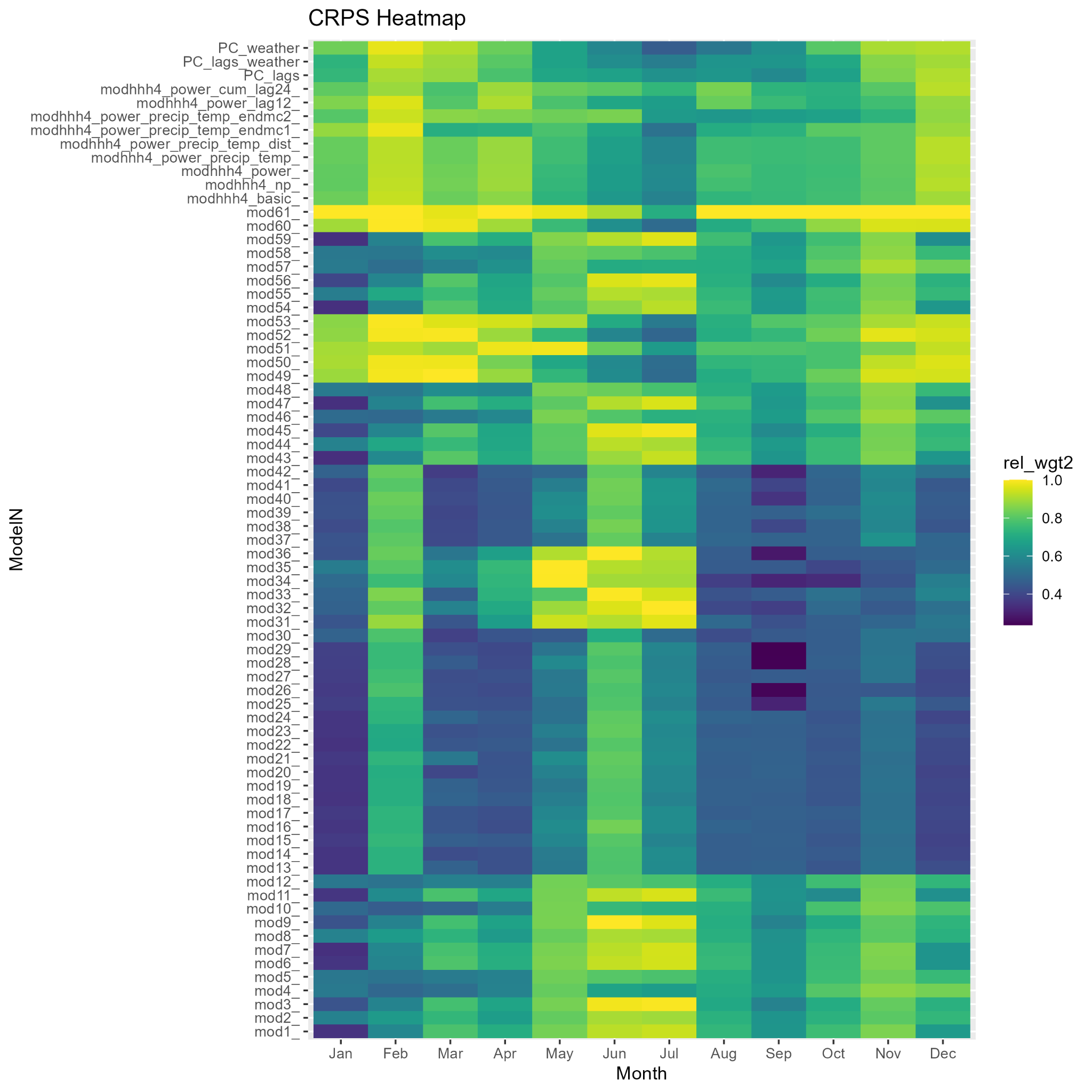


Fig C: Heat map of CRPS relative values, with the x-axis representing months and the y-axis showing the tested models; each cell shows a model’s CRPS divided by the mean CRPS for that month.

**
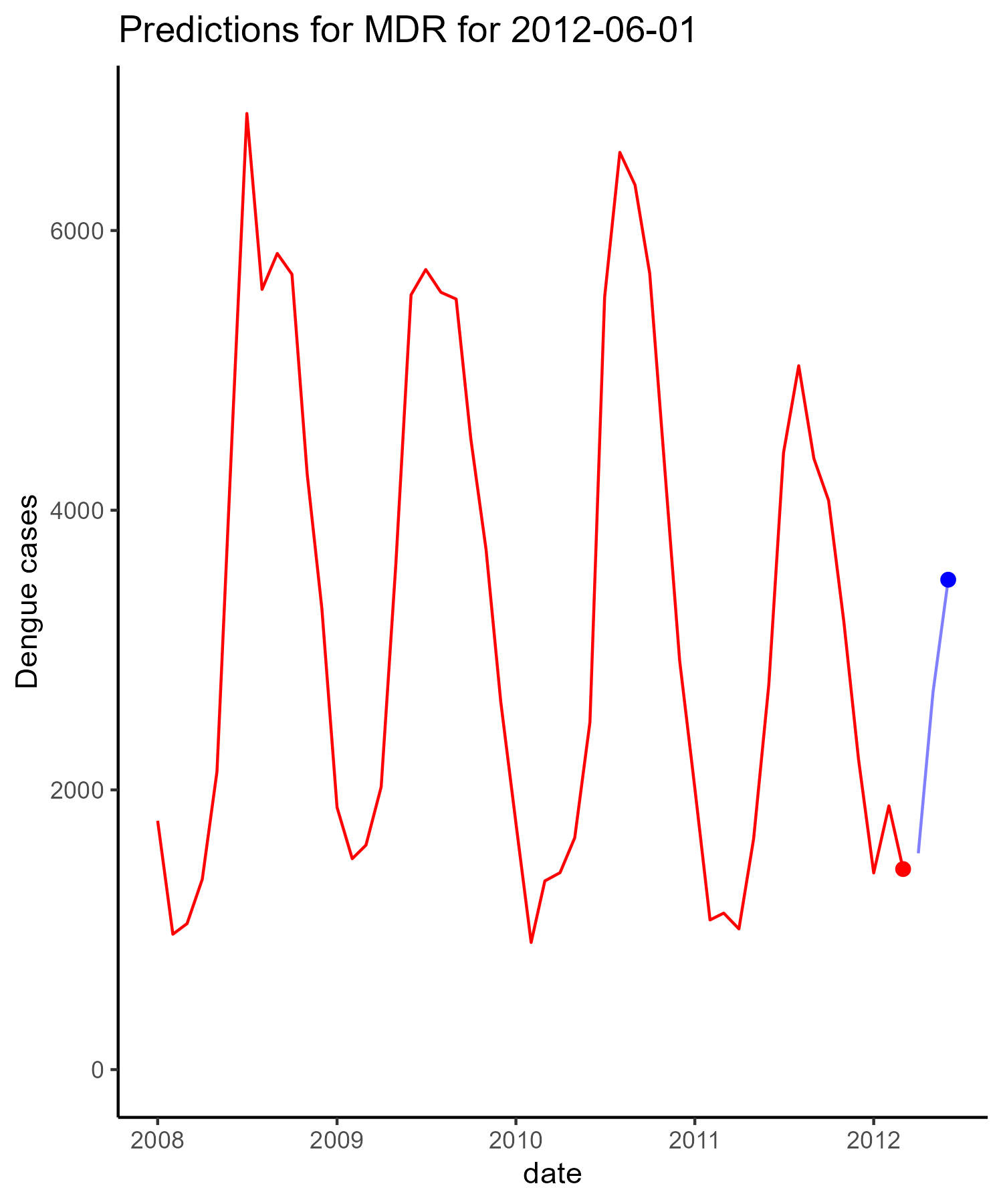

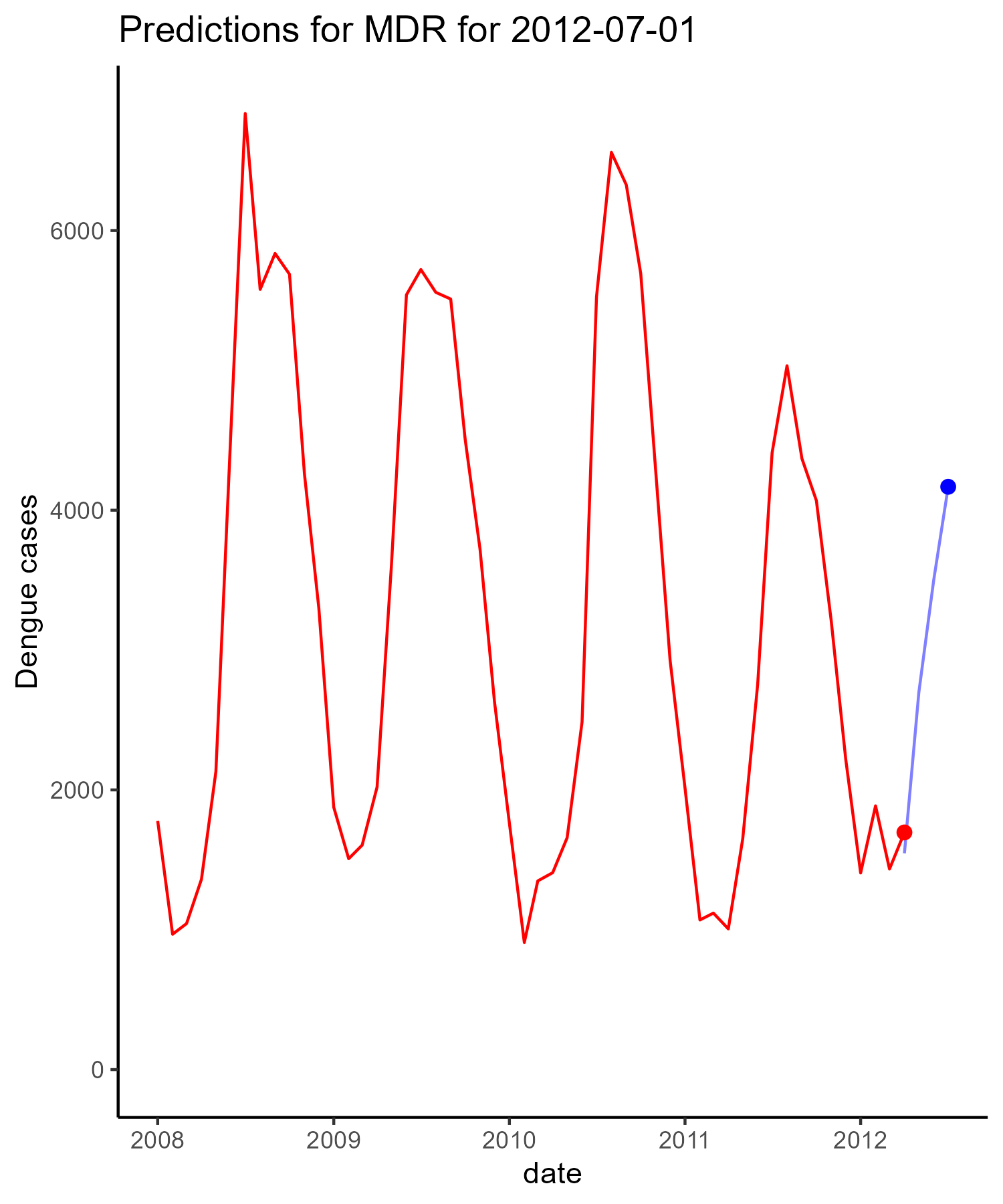
**

**
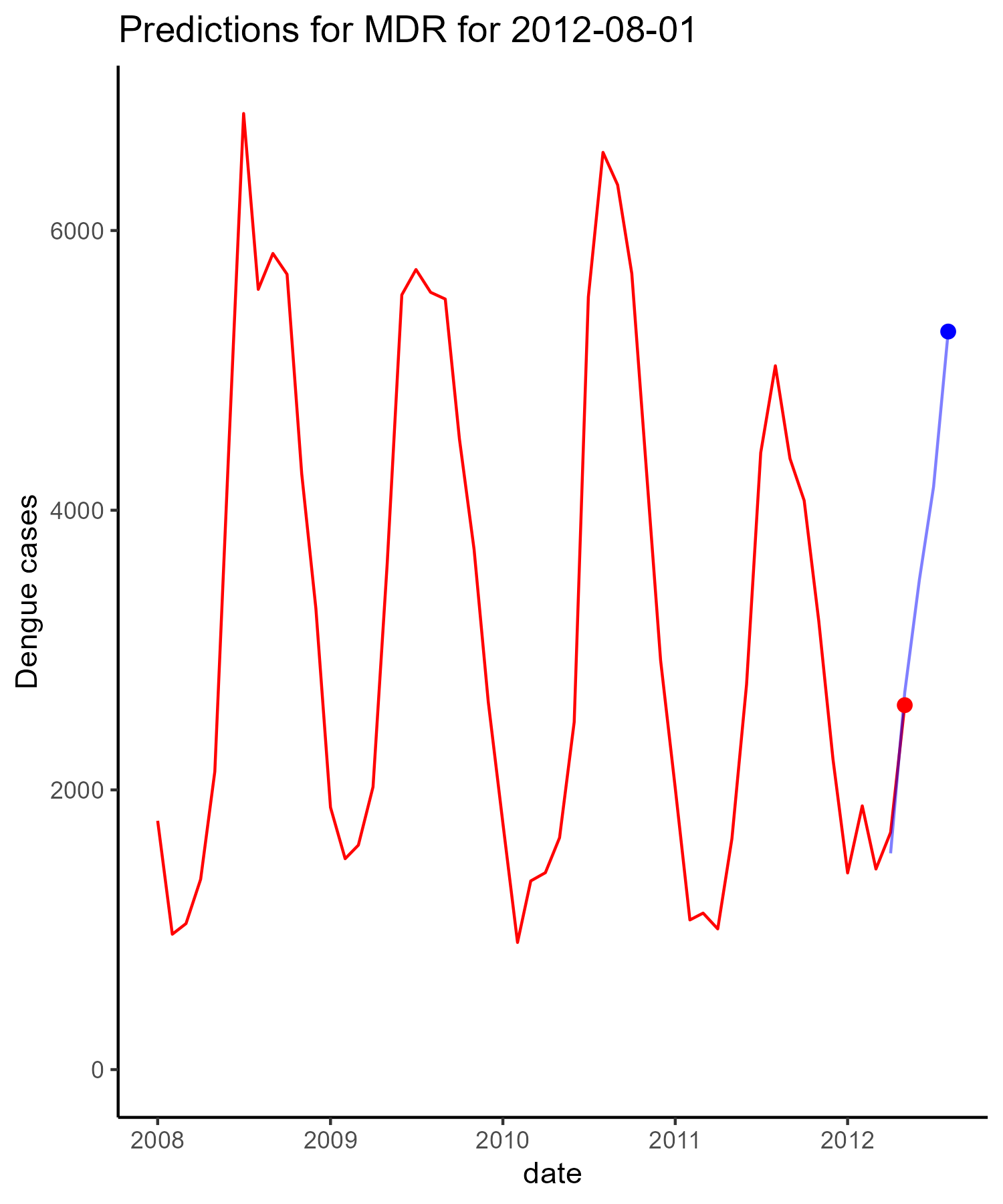

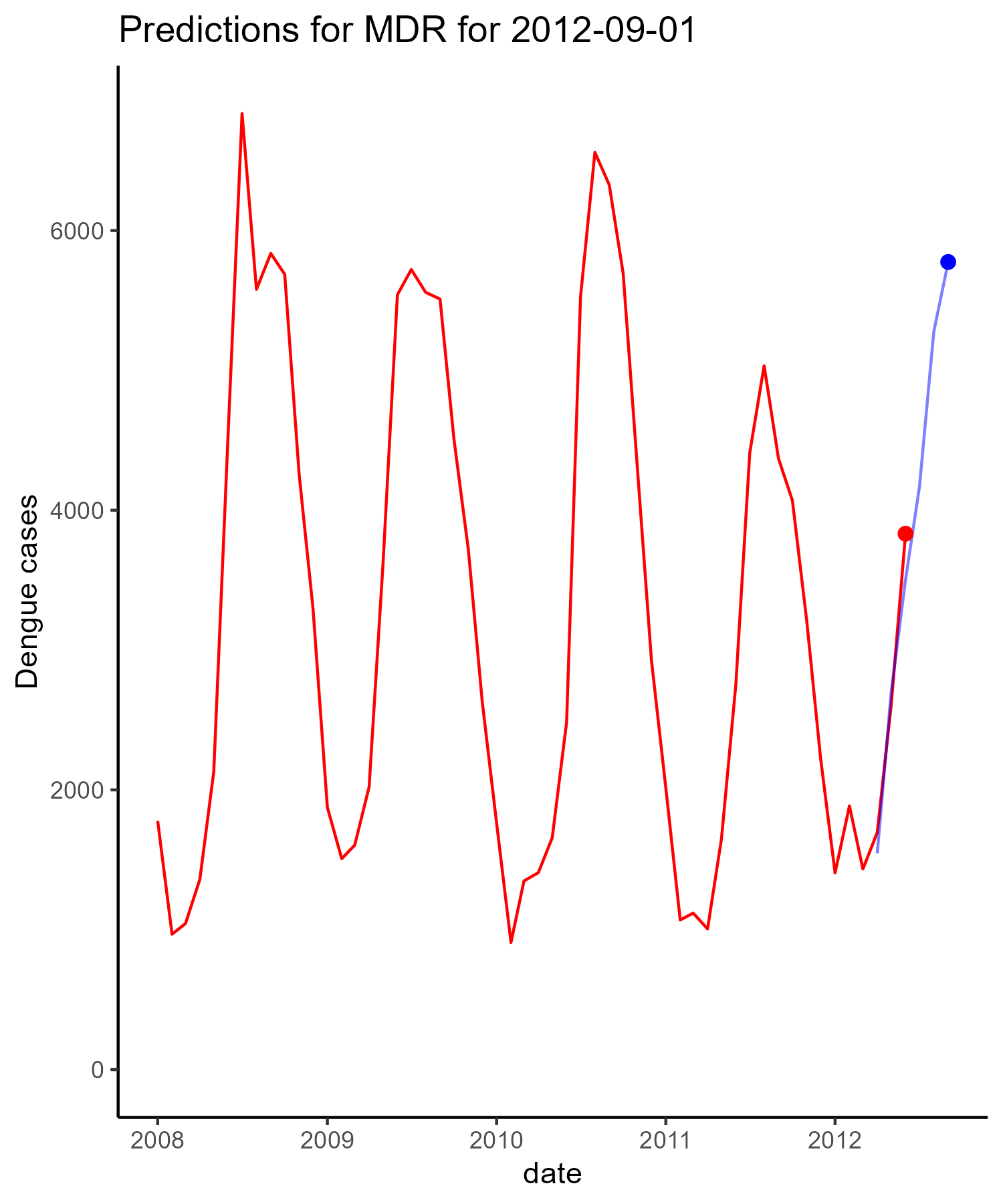
**

Fig D: 3-month ahead predictions of dengue cases for the Mekong Delta Region using the ensemble model. The red lines represent observed dengue cases, while the blue lines denote predicted cases. The forecasts demonstrate the model's ability to capture seasonal trends and predict future outbreaks.


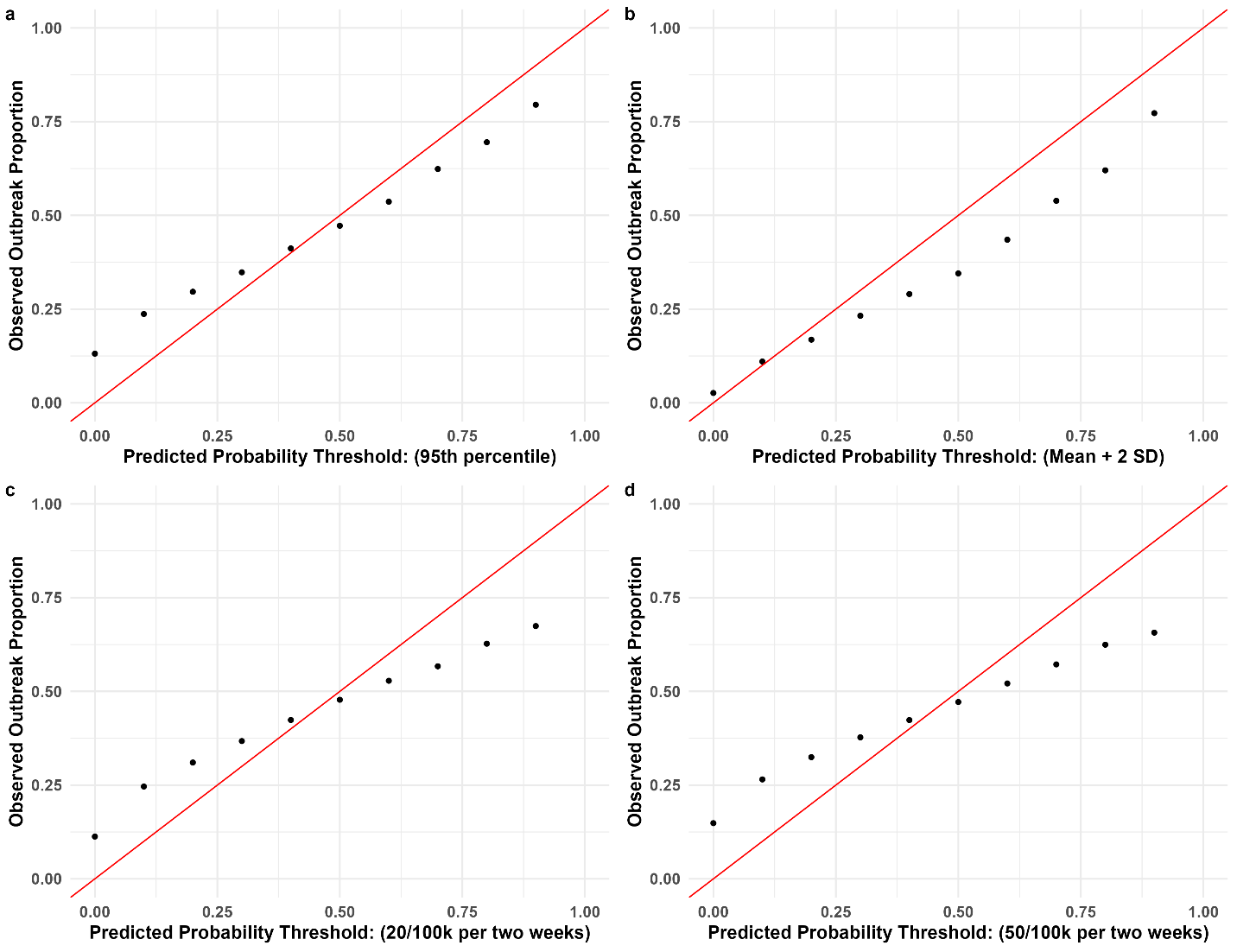


Fig E: Accuracy of the ensemble model for selected outbreak thresholds based on the probability of predicting an outbreak compared to the observed outbreak proportion, Mekong Delta Region, 2012-2016. X-axis represents bins of predicted probabilities (0-<0.1, 0.1-<0.2, 0.2-<0.3…0.9-1). The Y-axis represents the proportion of observations for which the observed exceeded the predicted.


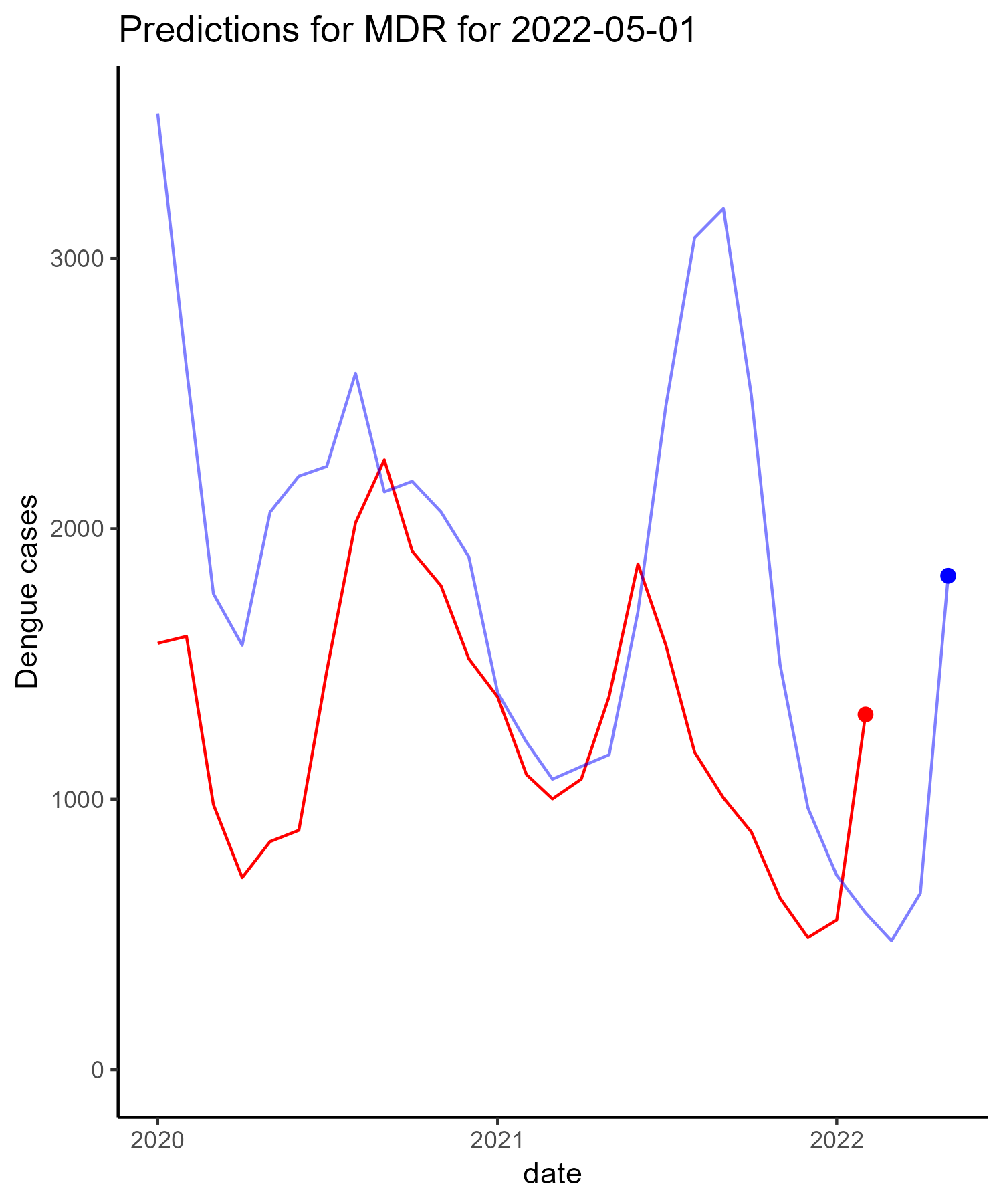

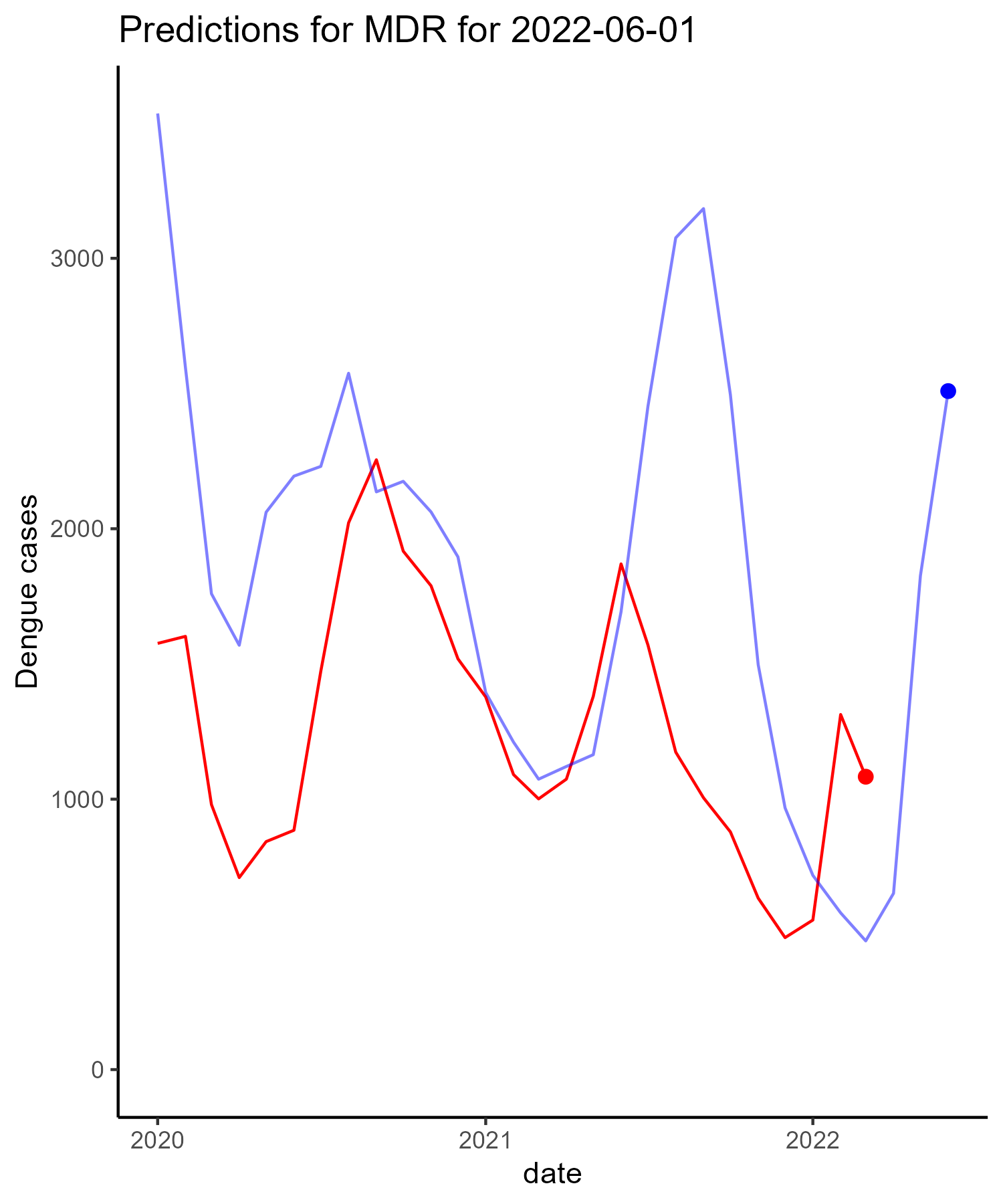


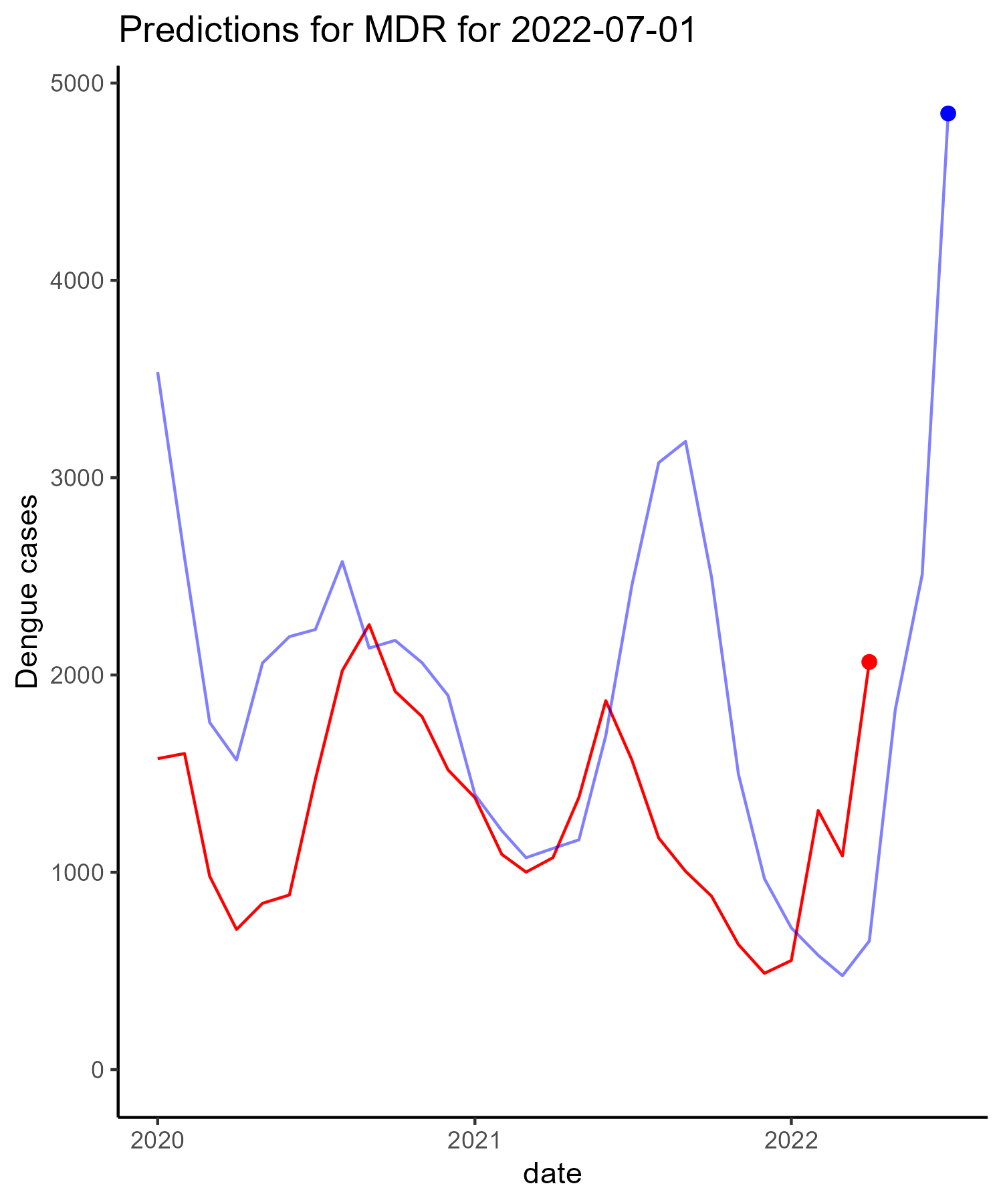

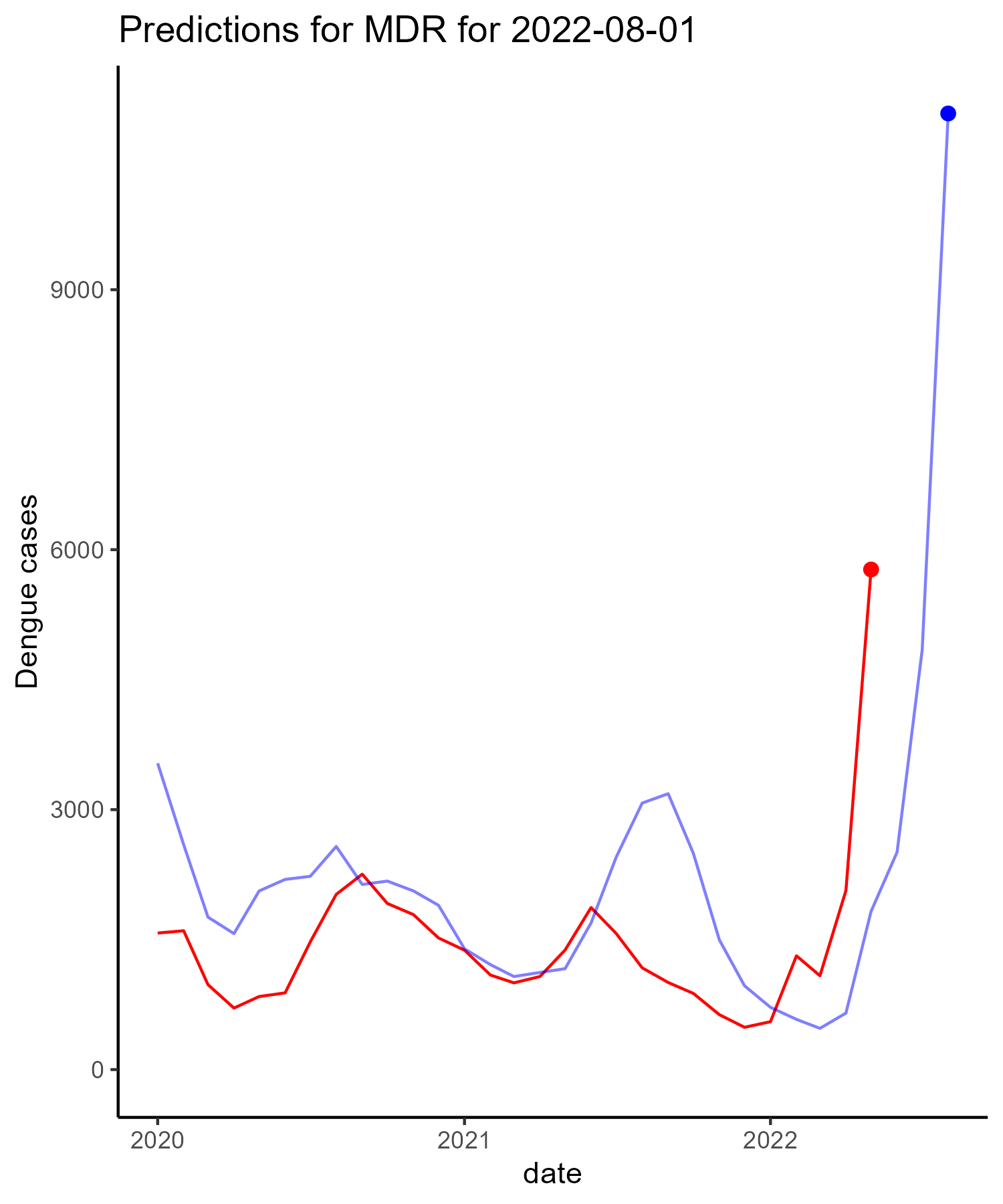


Fig F: 3-month ahead out-of-sample (2017-2022) predictions of dengue cases using the ensemble model for the Mekong Delta Region. The red lines represent observed dengue cases, while the blue lines denote predicted cases. The forecasts demonstrate the model's ability to capture seasonal trends and predict future outbreaks.

*
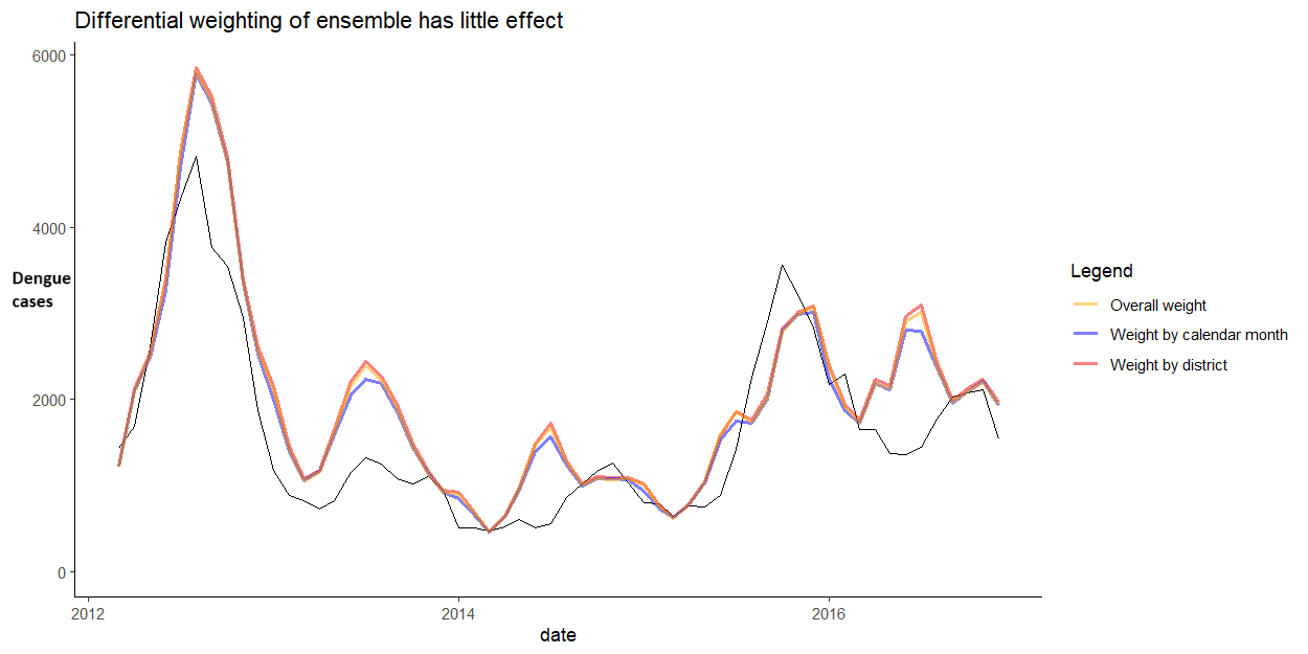
*

Fig G: Observed dengue cases and the ensemble predictions using overall, monthly, or district weights. The coloured lines almost overlap, showing that different weights change the forecast very little.


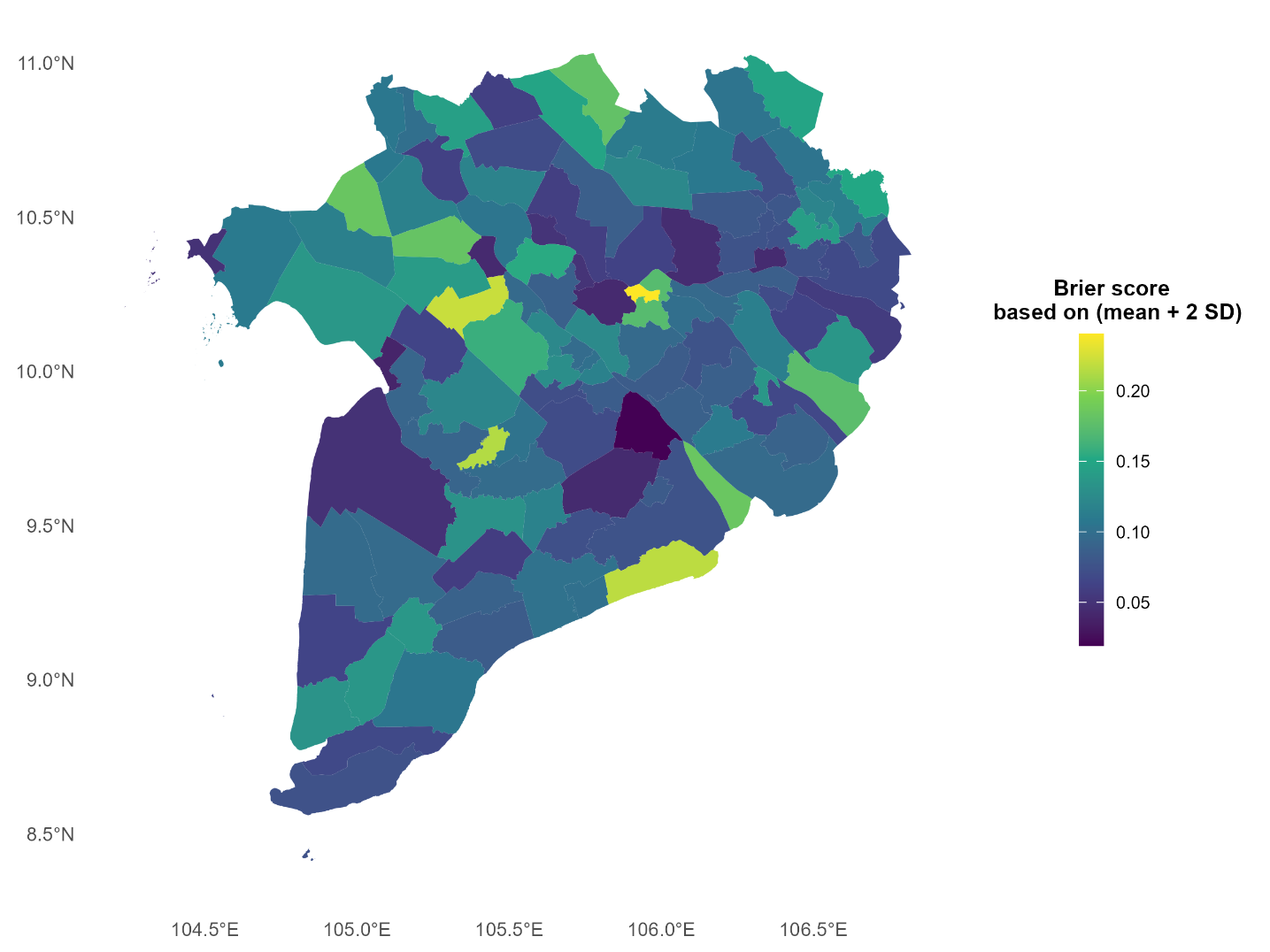


*Fig H: District‐level Brier scores (Mean + 2 SD threshold model). Each district is shaded according to its Brier score (darker purple = lower Brier, better predictive accuracy; bright yellow = higher Brier, less accuracy. Base map shapefiles sourced from DIVA-GIS (*[*https://diva-gis.org/data.html*](https://diva-gis.org/data.html)*), originally from GADM (*[*https://gadm.org/*](https://gadm.org/)*), under CC BY 4.0 license.*
