# Supplementary material for: A district-level ensemble model to enhance dengue prediction and control for the Mekong Delta Region of Vietnam
Source: PLoS Negl Trop Dis. 2025 Sep 29;19(9):e0013571. doi: 10.1371/journal.pntd.0013571 (PMC12507206; doi:10.1371/journal.pntd.0013571)
Supplement: S2 — (DOCX) [file pntd.0013571.s002.docx]

**S2: Three-Month-Ahead Dengue Cases Forecasts vs. Observations**

Three-month ahead forecasts of dengue cases across districts, compared to the observed cases, using different dates. Panels display predictions from three models of the spatio-temporal model, the PCA-based model, and the hhh4 model, along with observed cases. The continuous colour scale indicates cases from low to high and is attached below (Fig A-Fig E)


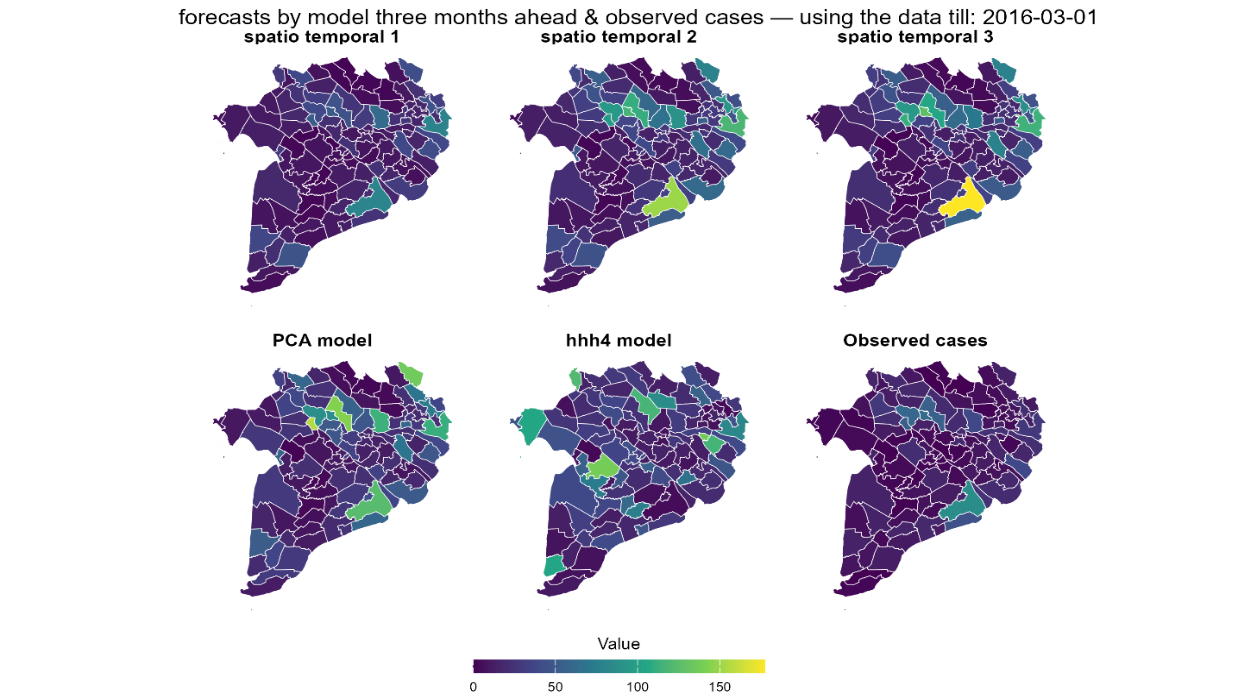


*Fig A: Three-month-ahead forecasts from five models for June 2016 (using data up to March 2016) compared with observed dengue cases*. *Base map shapefiles sourced from DIVA-GIS (*[*https://diva-gis.org/data.html*](https://diva-gis.org/data.html)*), originally from GADM (*[*https://gadm.org/*](https://gadm.org/)*), under CC BY 4.0 license.*


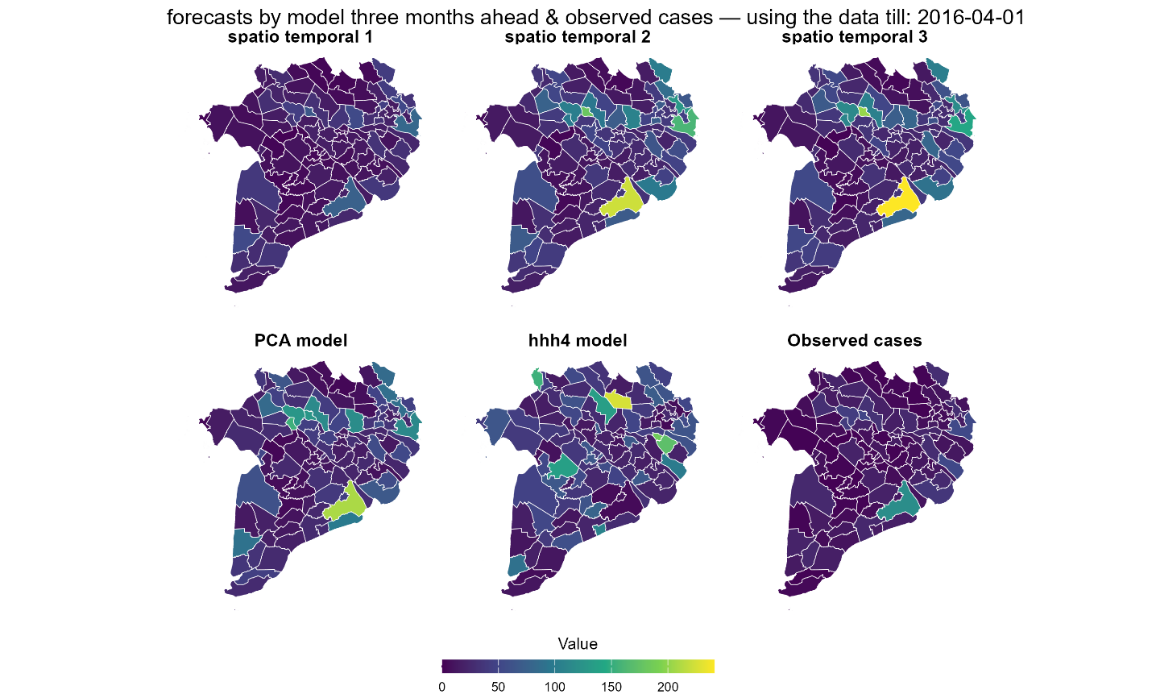


*Fig B: Three-month-ahead forecasts from five models for July 2016 (using data up to April 2016) compared with observed dengue cases*. *Base map shapefiles sourced from DIVA-GIS (*[*https://diva-gis.org/data.html*](https://diva-gis.org/data.html)*), originally from GADM (*[*https://gadm.org/*](https://gadm.org/)*), under CC BY 4.0 license.*


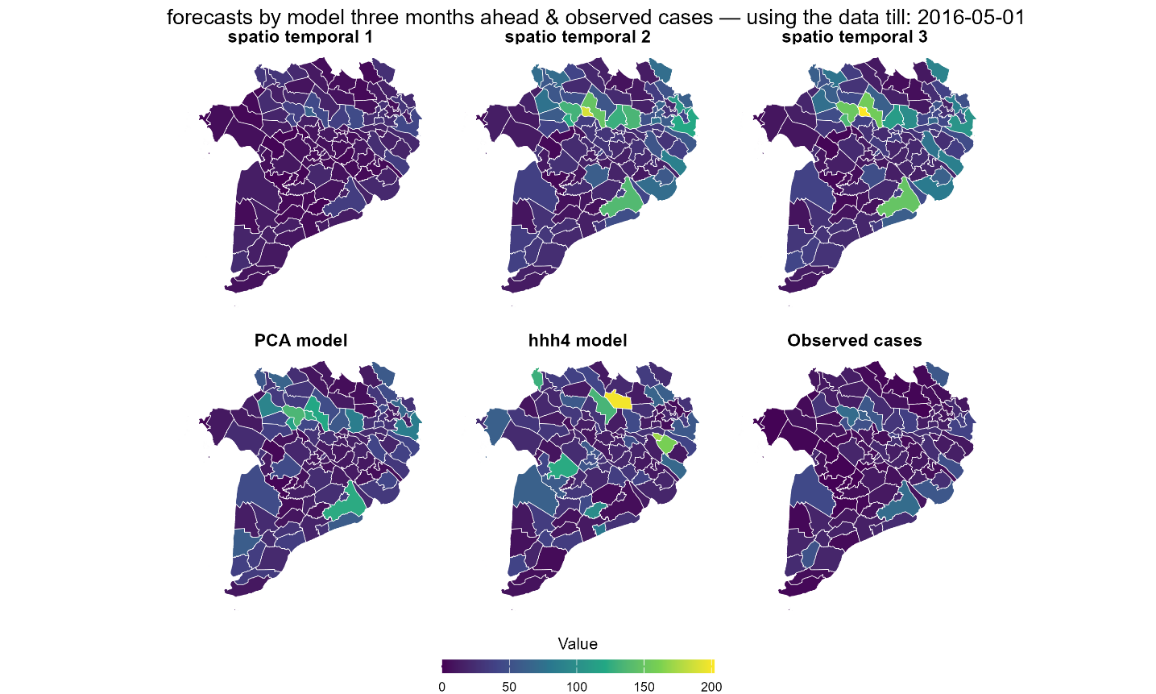


*Fig C: Three-month-ahead forecasts from five models for August 2016 (using data up to May 2016) compared with observed dengue cases.* *Base map shapefiles sourced from DIVA-GIS (*[*https://diva-gis.org/data.html*](https://diva-gis.org/data.html)*), originally from GADM (*[*https://gadm.org/*](https://gadm.org/)*), under CC BY 4.0 license.*


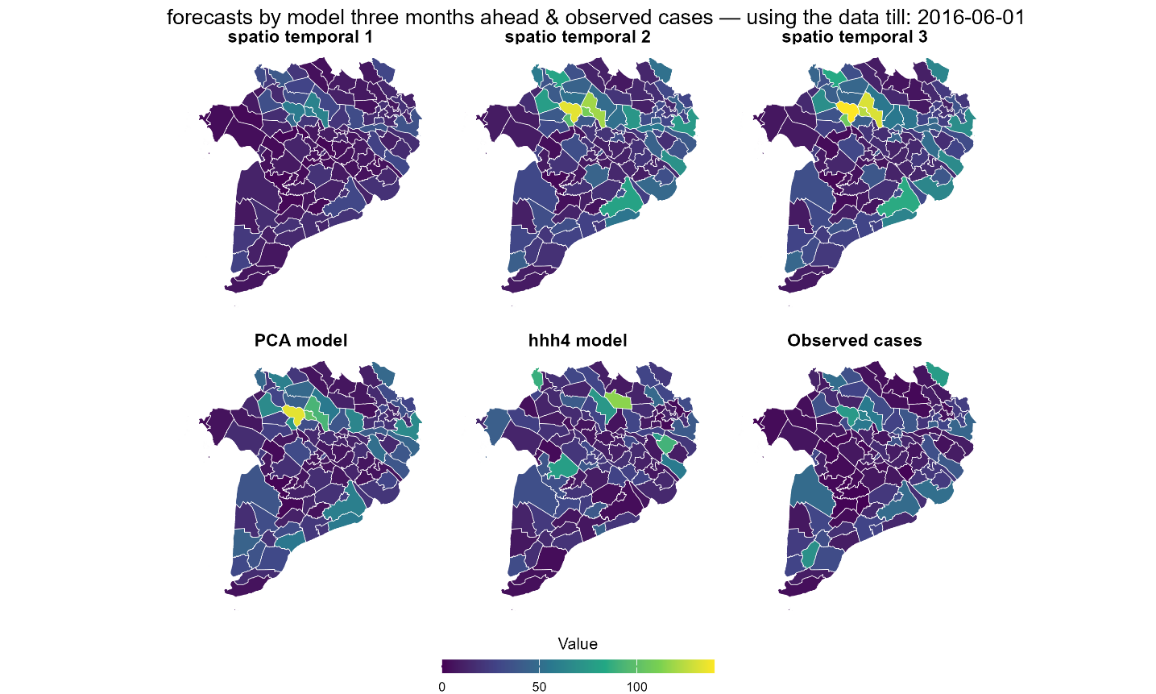


*Fig D: Three-month-ahead forecasts from five models for September 2016 (using data up to June 2016) compared with observed dengue cases.* *Base map shapefiles sourced from DIVA-GIS (*[*https://diva-gis.org/data.html*](https://diva-gis.org/data.html)*), originally from GADM (*[*https://gadm.org/*](https://gadm.org/)*), under CC BY 4.0 license.*


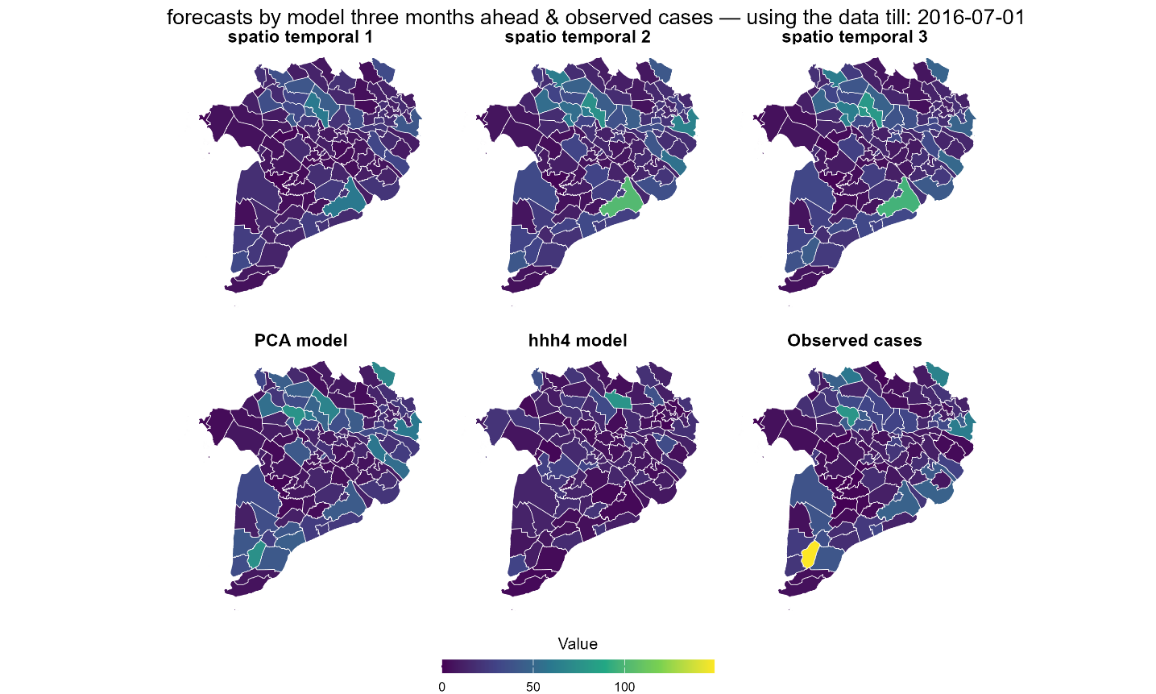


*Fig E: Three-month-ahead forecasts from five models for October 2016 (using data up to July 2016) compared with observed dengue cases*. *Base map shapefiles sourced from DIVA-GIS (*[*https://diva-gis.org/data.html*](https://diva-gis.org/data.html)*), originally from GADM (*[*https://gadm.org/*](https://gadm.org/)*), under CC BY 4.0 license.*

A Comparison of observed versus predicted outbreak flags. Observed outbreaks are identified using the threshold (Mean + 2 SD) of dengue for the same month over the previous five years, excluding high epidemic years, while predicted outbreaks are derived from our ensemble forecasting model and are attached below (S2 Fig6 S2 Fig9)


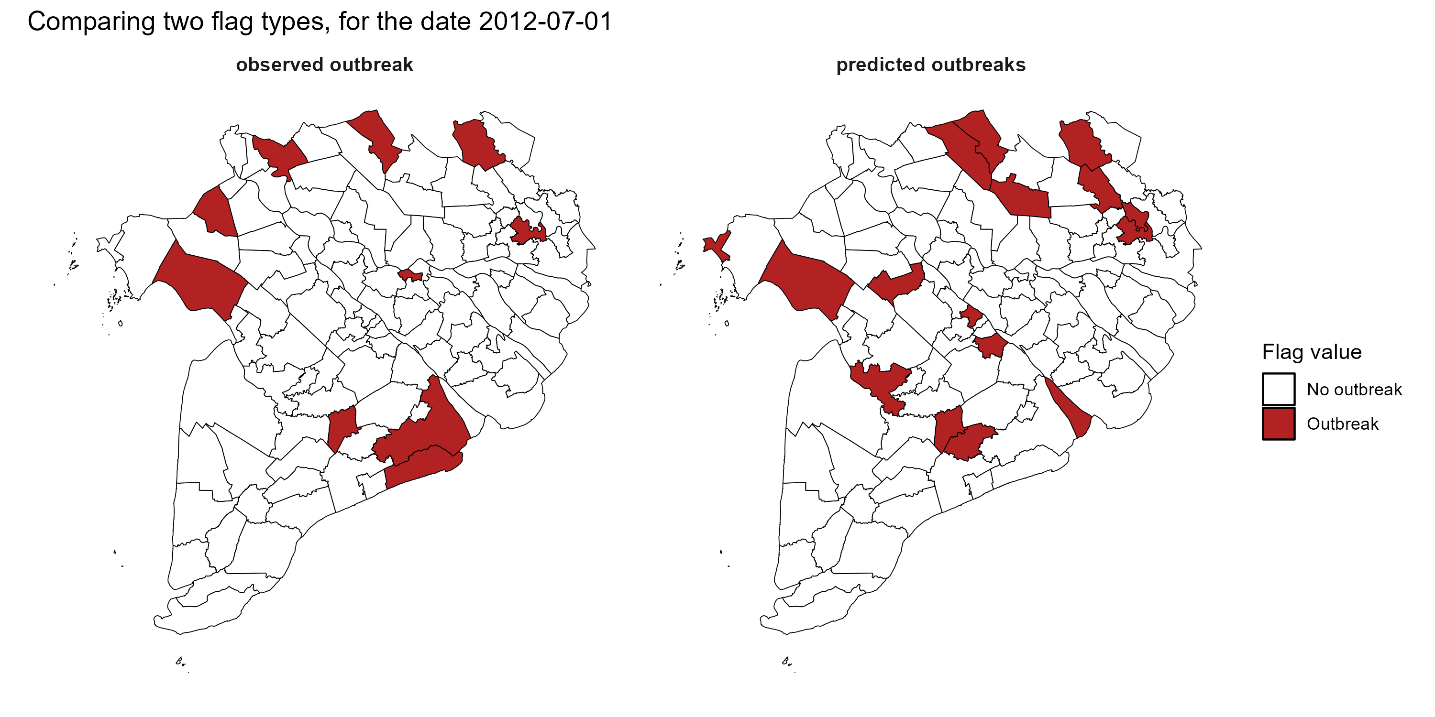


*Fig F: Comparison of ensemble‐predicted outbreaks for July 2012 with observed outbreaks defined by the Mean + 2 SD threshold*. *Base map shapefiles sourced from DIVA-GIS (*[*https://diva-gis.org/data.html*](https://diva-gis.org/data.html)*), originally from GADM (*[*https://gadm.org/*](https://gadm.org/)*), under CC BY 4.0 license.*


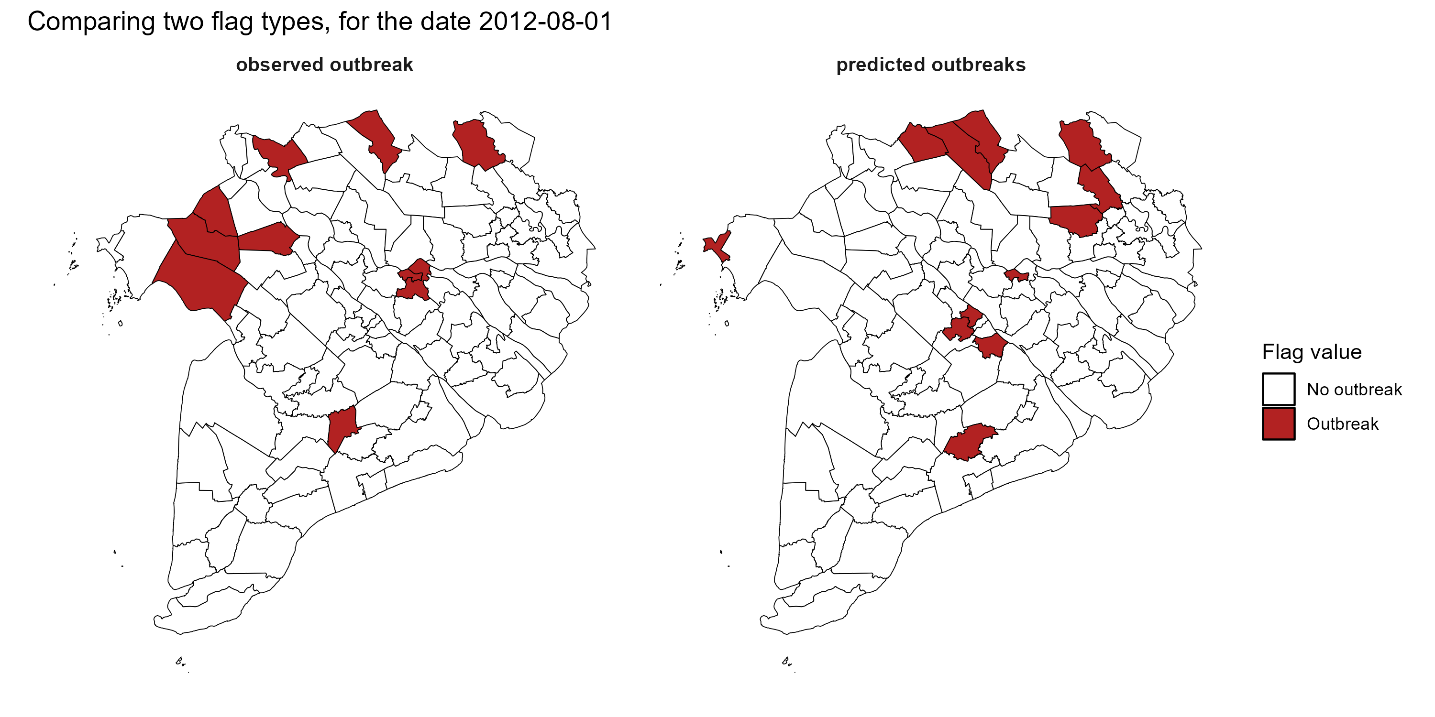


*Fig G: Comparison of ensemble‐predicted outbreaks for August 2012 with observed outbreaks defined by the Mean + 2 SD threshold*. *Base map shapefiles sourced from DIVA-GIS (*[*https://diva-gis.org/data.html*](https://diva-gis.org/data.html)*), originally from GADM (*[*https://gadm.org/*](https://gadm.org/)*), under CC BY 4.0 license.*


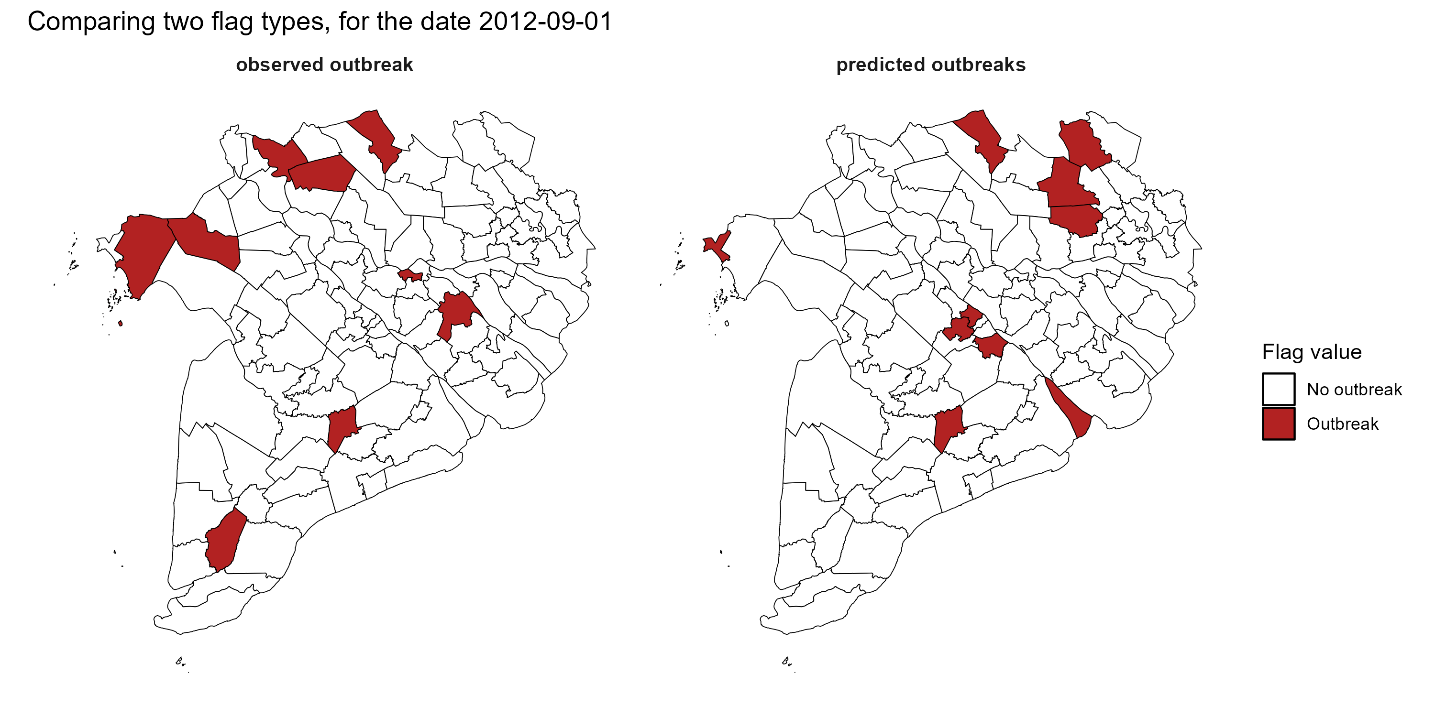


*Fig H: Comparison of ensemble‐predicted outbreaks for September 2012 with observed outbreaks defined by the Mean + 2 SD threshold*. *Base map shapefiles sourced from DIVA-GIS (*[*https://diva-gis.org/data.html*](https://diva-gis.org/data.html)*), originally from GADM (*[*https://gadm.org/*](https://gadm.org/)*), under CC BY 4.0 license.*


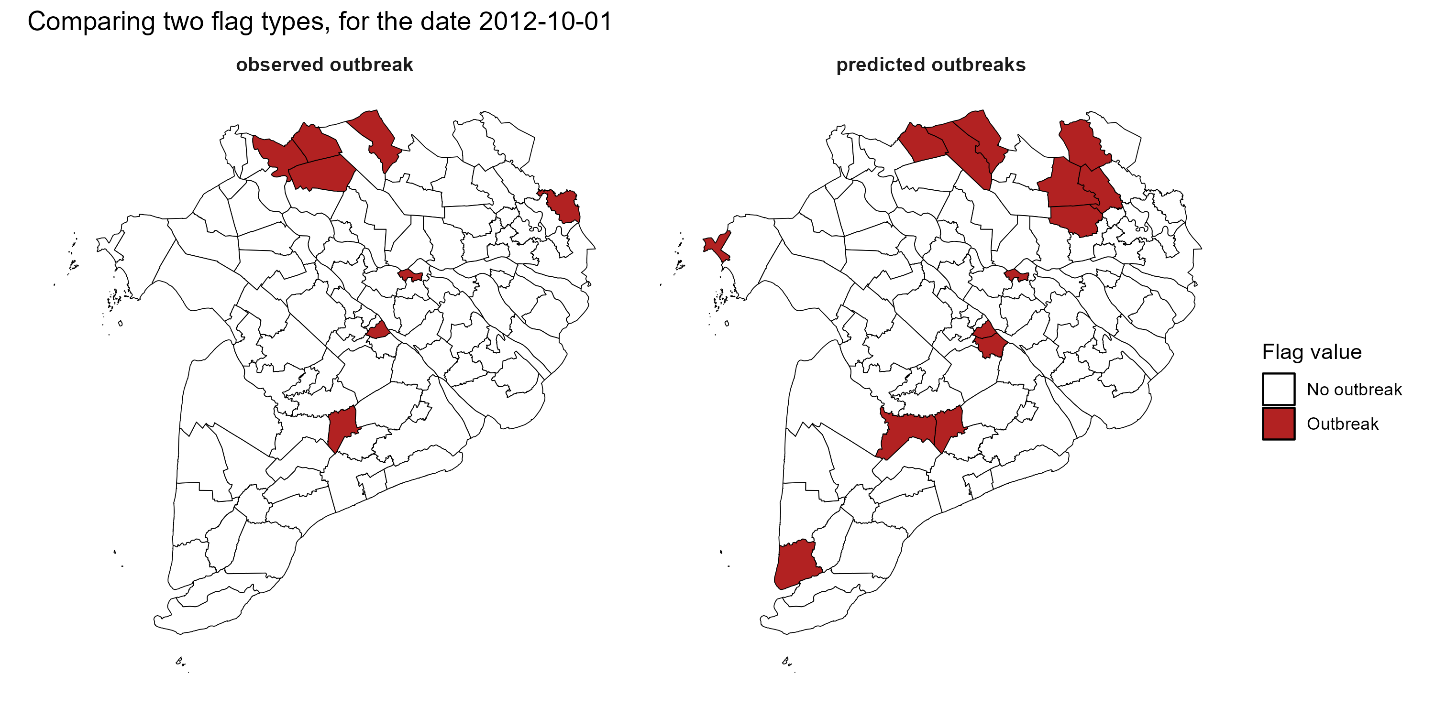


*Fig I: Comparison of ensemble‐predicted outbreaks for October 2012 with observed outbreaks defined by the Mean + 2 SD threshold*. *Base map shapefiles sourced from DIVA-GIS (*[*https://diva-gis.org/data.html*](https://diva-gis.org/data.html)*), originally from GADM (*[*https://gadm.org/*](https://gadm.org/)*), under CC BY 4.0 license.*
