# Supplementary material for: A district-level ensemble model to enhance dengue prediction and control for the Mekong Delta Region of Vietnam
Source: PLoS Negl Trop Dis. 2025 Sep 29;19(9):e0013571. doi: 10.1371/journal.pntd.0013571 (PMC12507206; doi:10.1371/journal.pntd.0013571)
Supplement: S3 — (DOCX) [file pntd.0013571.s003.docx]

**S3: Spatiotemporal and Forecasting Methods**

**Hierarchical Bayesian spatiotemporal models**

Let $Y_{i,t}$ be the number of dengue cases in the district $i=1,2,..,n$ and time $t=1,2,..,T,$ where $n$ is the total number of districts in the data set, and $T$ is the total number of time steps for which the model is fitted. The models used a Poisson likelihood when fitting to the number of observed cases. The generic model is defined as:

$$Y_{i,t}\sim Poisson(\mu_{i,t}\mathbf{)}$$

$$log\left( \frac{\mu_{i,t}}{\left( p_{i,a\left[ t \right]} \right)} \right)=\alpha+log\left( {(Y}_{i,t-3}+1)/\left( p_{i,a\left[ t \right]} \right) \right)+\sum_{k} \beta_{k}X_{k,i,t}+\eta_{i,m\left[ t \right]}+\delta_{i,a[t]}+\theta_{i}$$

Where

- $Y_{i,t-3}$ is the lagged number of monthly dengue cases in the district$i$ and time $t$ lagged 3 months.
- $\alpha$ is the intercept.
- $p_{i,a\left[ t \right]}$: is the population for district i and year $a\left[ t \right]$, included as an offset to adjust case counts by population.
- $\beta_{k}X_{k,i,t}$: represents the fixed effects for the covariates, with $\beta_{k}$​ being the corresponding coefficients.
- $\delta_{i,a[t]}$ is a random effect with a first order autoregressive structure that captures temporal variation. In some models, the random effect is the same across districts, while in other models, there was a separate AR(1) random effect for each district, with hyperparameters (e.g., variance) shared across districts.
- $\eta_{i,m\left[ t \right]}$ : is seasonality which has a cyclic 1st order random walk process for time, with a separate random walk estimated for each district but with a shared hyperparameter on the variance.
- $\theta_{i}$: is a random effect for each district that accounts for local variability that is unique to the district (unstructured noise), as well as the influence of neighbouring districts (spatial correlation), modelled by some models like Besag-York-Mollie.

Delayed effects of meteorological factors were accounted for by a lag of 3 months. Normal priors were set for the fixed effects, with a mean of 0 and a variance of 1, while the global intercept had a prior with a mean of 0 and a variance of 25. Spatial dependencies were modelled using the Besag model, with a log-gamma prior for precision to ensure moderate smoothness (log-gamma(1,1). Penalising Complexity (PC) priors were applied across all random effects. The BYM and BYM2 models incorporated spatial and district-specific random effects, using PC priors to encourage smoother spatial variation (BYM/BYM2 precisions: P(σ > 1) = 0.01, i.e., u = 1, α = 0.01; BYM2 mixing parameter: u = 0.5, α = 0.5). Temporal autocorrelation was modelled using an AR(1) process, with PC priors applied to both the temporal and autocorrelation parameters (AR(1) marginal SD: P(σ > 0.5) = 0.01, i.e., u = 0.5, α = 0.01; correlation prior pc.cor0 with P(|ρ| > 0.5) = 0.75). For unstructured random effects, PC priors were used to capture district-specific variability (iid: P(σ > 1) = 0.01, i.e., u = 1, α = 0.01). Seasonal trends were modelled using an RW1 process (Random Walk of Order 1 with PC prior on the precision: P(σ > 0.3) = 0.01, i.e., u = 0.3, α = 0.01 and is a commonly used model in Bayesian statistics for smoothing temporal or spatial data ), with PC priors providing flexibility in the smoothness of monthly effects. Models were fitted in R version 4.2.2 using the *INLA* package.

**hhh4 models**

This model predicts the number of dengue cases in a district $i$at time $t$ autocorrelation, and spatial spread. Each of these components—endemic, epidemic, and spatial—are modeled as a function of covariates. The outcome $Y_{i,t}$representing the observed dengue cases follows a Negative Binomial distribution:

$$Y_{i,t}\sim NegBin(\mu_{i,t,} \psi)$$

Where$\mu_{i,t,}$​ is the mean and $\psi$controls the dispersion. The model decomposes $\mu_{i,t,}$as:

$$\mu_{i,t}\mathbf{=}v_{i,t}+\lambda_{i,t}Y_{i,t-1}+\phi_{i,t}\sum_{j\neq i} w_{j,i}Y_{j,t-1}$$

**Endemic Component** $v_{i,t}$

This term captures seasonality and the covariates effect

$$\log\left( v_{i,t} \right)=\alpha+\log\left( p_{i,a\left[ t \right]} \right)+\sin\left( \frac{2\pi t}{12} \right)+\cos\left( \frac{2\pi t}{12} \right)+\sum_{k} \beta_{k}X_{k,i,t}$$

**epidemic component (autoregressive) Component** $\lambda_{i,t}Y_{i,t-1}$**​:**

This component models the influence of past cases within the same district.

$log$*(*$\lambda_{i,t} Y_{i,t-1})=\alpha+ \sum_{k} \beta_{k}X_{k,i,t}$

**spatial spread component (neighbourhood) Component** $\phi_{i,t}\sum_{j\neq i} w_{j,i}Y_{j,t-1}$

This captures the influence of neighbouring districts’ past cases on the current district.

$log$($\phi_{i,t}$) = $\alpha+ \sum_{k} \beta_{k}X_{k,i,t}$

Where $\alpha is the$intercept, $p_{i,a\left[ t \right]}$ is the population offset for the district $i$at time $a[t]$, $\beta_{k}X_{k,i,t} is the$fixed covariates (e.g., 3-month lagged temperature and precipitation), $w_{j,i}$ represents the spatial weights, reflecting the influence of neighbouring districts through a power-law function, $\sin\left( \frac{2\pi t}{12} \right)\mathrm{and}\cos\left( \frac{2\pi t}{12} \right)$ are the seasonal harmonic terms to capture seasonality over a 12-month cycle.

Models were fitted in R version 4.2.2 using the surveillance package ([27](#_ENREF_27)).

**Supervised (“Y-aware”) principal components regression**

For each district $i$at time $t$, the dengue incidence $Y_{i,t}$ is modelled as a function of multiple covariates and lagged predictors. The covariate matrix $X$ includes standardised lagged covariates for all districts ([29](#_ENREF_29)).

A univariate linear regression is performed between the log-transformed dengue case counts and each covariate $X_{k}$to calculate the regression slope:

$$\log\left( Y_{i,t} \right)=\beta_{k} X_{k,i,t}+\varepsilon_{i,t}$$

Where $\beta_{k}$​is the regression coefficient for the covariate $k$. $X_{k,i,t}$ is the value of the covariate $k$ for district $i$at time $t, \mathrm{and} \varepsilon_{i,t}$ is the error term accounting for unexplained variability.

Each covariate is then multiplied by its corresponding slope:

$$X_{k,i,t}^{'}{=\beta}_{k} X_{k,i,t}-mean(\beta_{k} X_{k,i,t})$$

After rescaling, principal components analysis is applied to the matrix $X'$ to extract the principal components (PCs). These PCs are new variables that are linear combinations of the original covariates, capturing the directions of maximum variance.

$${PC}_{p}=\sum w_{p,k}X_{k,i,t}^{'}$$

- ${PC}_{p}$​ is the value of the $p$-th principal component.
- $w_{p,k}$ is the weight (loading) of covariate k in the $p$-th principal component.

The top 10 components, which explain (80%-90%) of the variance, were retained for further analysis.

The final regression model predicts dengue incidence by combining the PCs, seasonal components, and an autoregressive term.

$$\log\left( \mu_{i,t} \right)=\alpha+\log\left( p_{i,a\left[ t \right]} \right)+\sum_{p=1}^{P} \beta_{p}{PC}_{p}+sin \left( \frac{2\pi t}{12} \right)+\cos\left( \frac{2\pi t}{12} \right)+\sum_{k} \beta_{k}X_{k,i,t}+\delta_{i,a[t]}$$

where:

- $\mu_{i,t}:$ is the expected number of cases in the district $i$ at time$t$.
- $\alpha$: is the intercept representing the baseline level of dengue incidence.
- ${log(p}_{i,a\left[ t \right])}:$ is the population offset for the district $i.$
- $\sum_{p=1}^{P} \beta_{p}{PC}_{p}$: is the sum of the principal components, each weighted by its coefficient $\beta_{p}.$
- $\sin\left( \frac{2\pi t}{12} \right)\mathrm{and}\cos\left( \frac{2\pi t}{12} \right) are the$seasonal effects using sine and cosine terms.
- $\delta_{i,a[t]}$ is the AR (1) random effect capturing temporal correlations.

Models were fitted in R version 4.2.2 using the INLA package.

**Build The Ensemble**Our ensemble is a weighted average of five statistical forecasting models chosen after testing 72 candidate models over the 2012–2016 TSCV period. it was constructed as follows:

1. Model testing

We defined 72 distinct model specifications (combinations of covariates, random‐effect structures, and space–time interaction terms).

For each of the 60 monthly “vintage dates” between 2012 and 2016, we refit all 72 models and computed three metrics on hold‐out data: CRPS (continuous ranked probability score), Bias (mean signed error), and diffuseness

2. Selecting top candidates

We examined each model’s performance at the monthly, annual, and district‐level scales.

We then performed a time‐series clustering of model performance profiles (i.e., CRPS curves over time). Models that clustered closely were deemed to behave similarly.

From those clusters, we chose the simplest model in each of the five best‐performing clusters, ensuring complementary strengths. This yielded our top five.

3. Assigning ensemble weights

For each of these five models, we computed its average over all months and districts.

We set each model’s weight (1/CRPS2)/sum(1/CRPS2).

Lower-CRPS models receive higher weight, but the squared inverse ensures that very small CRPS differences still produce modest weight differences, thereby avoiding over-concentration (see Fig. S7 in Section 1).

4. Generating final forecasts

For any new “vintage date” after 2016, we run all five chosen models, extract their posterior predictive distributions for each district and horizon, and then compute a weighted combination of those five distributions.
