# Supplementary material for: A district-level ensemble model to enhance dengue prediction and control for the Mekong Delta Region of Vietnam
Source: PLoS Negl Trop Dis. 2025 Sep 29;19(9):e0013571. doi: 10.1371/journal.pntd.0013571 (PMC12507206; doi:10.1371/journal.pntd.0013571)

**S4: District-Level Time Series of Dengue Cases and Environmental Covariates**

All below plots show monthly dengue counts alongside our four weather measures (humidity, temperature, rainfall, and wind), each scaled to have a mean of zero and unit variance. The plots show that dengue cases peak during the rainy, humid season and rise when temperatures are higher. Because every weather line is standardized, it’s easy to compare them directly and observe that increases in rainfall, humidity, and temperature align with higher dengue case numbers.


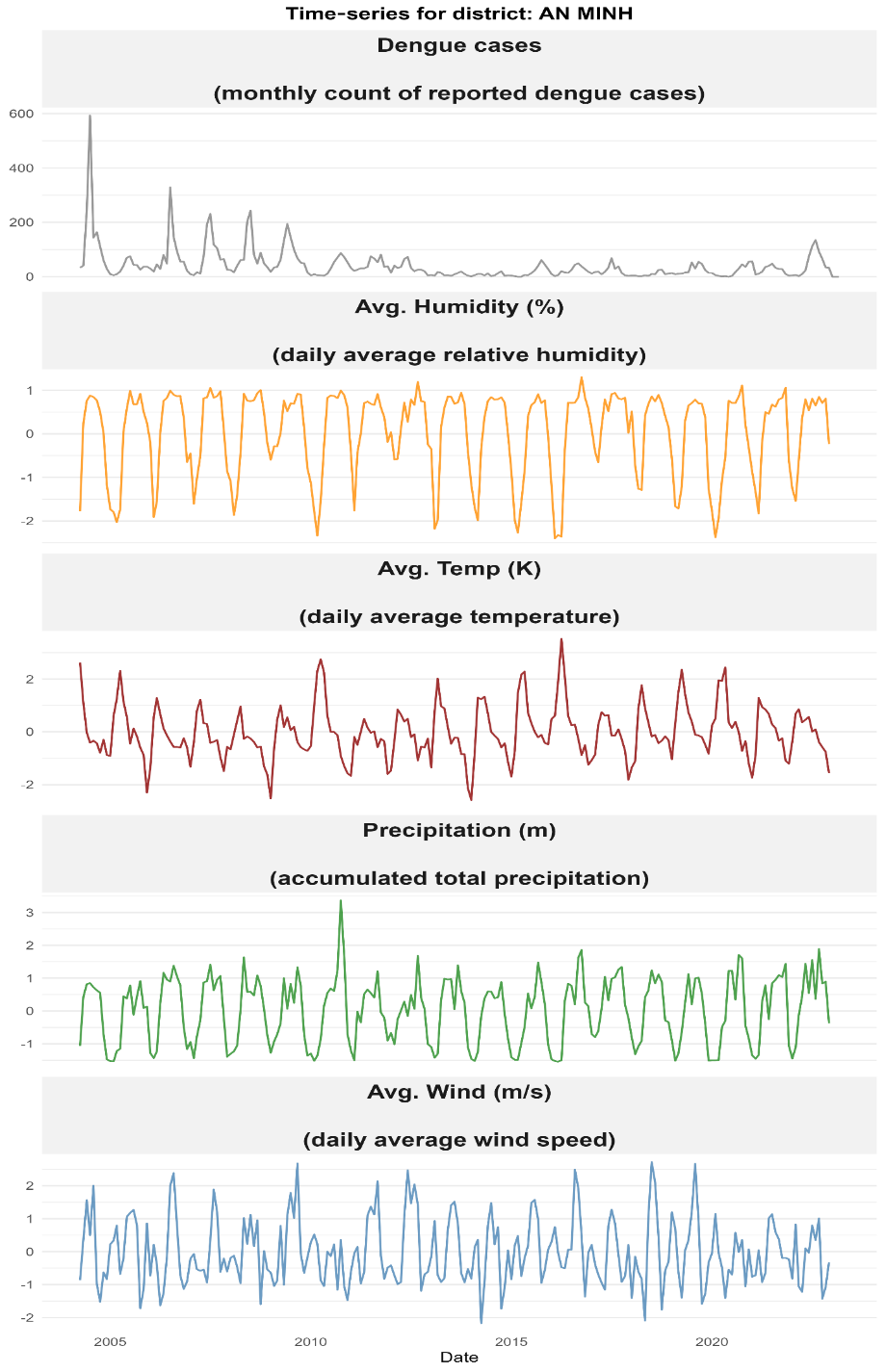


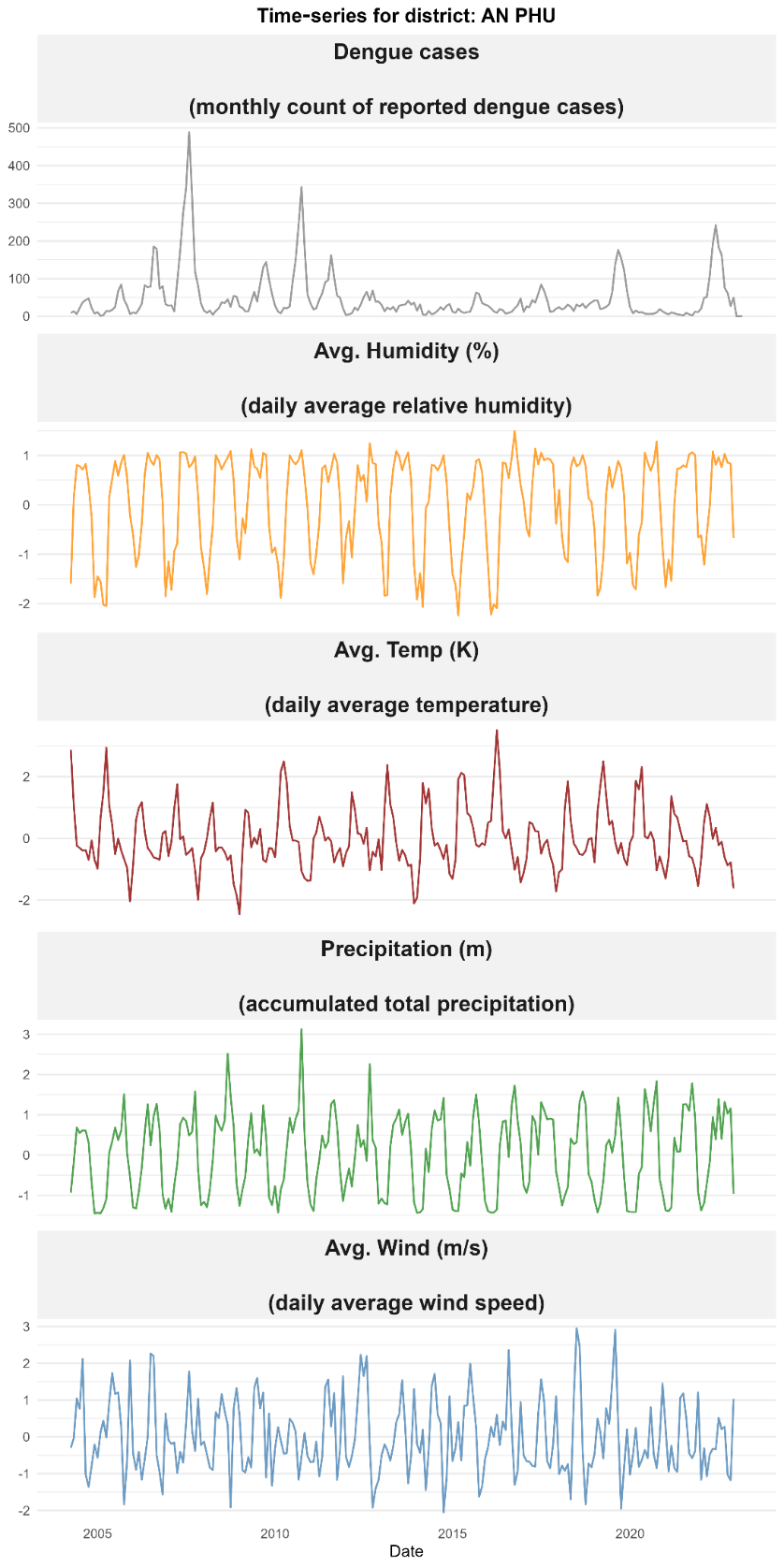

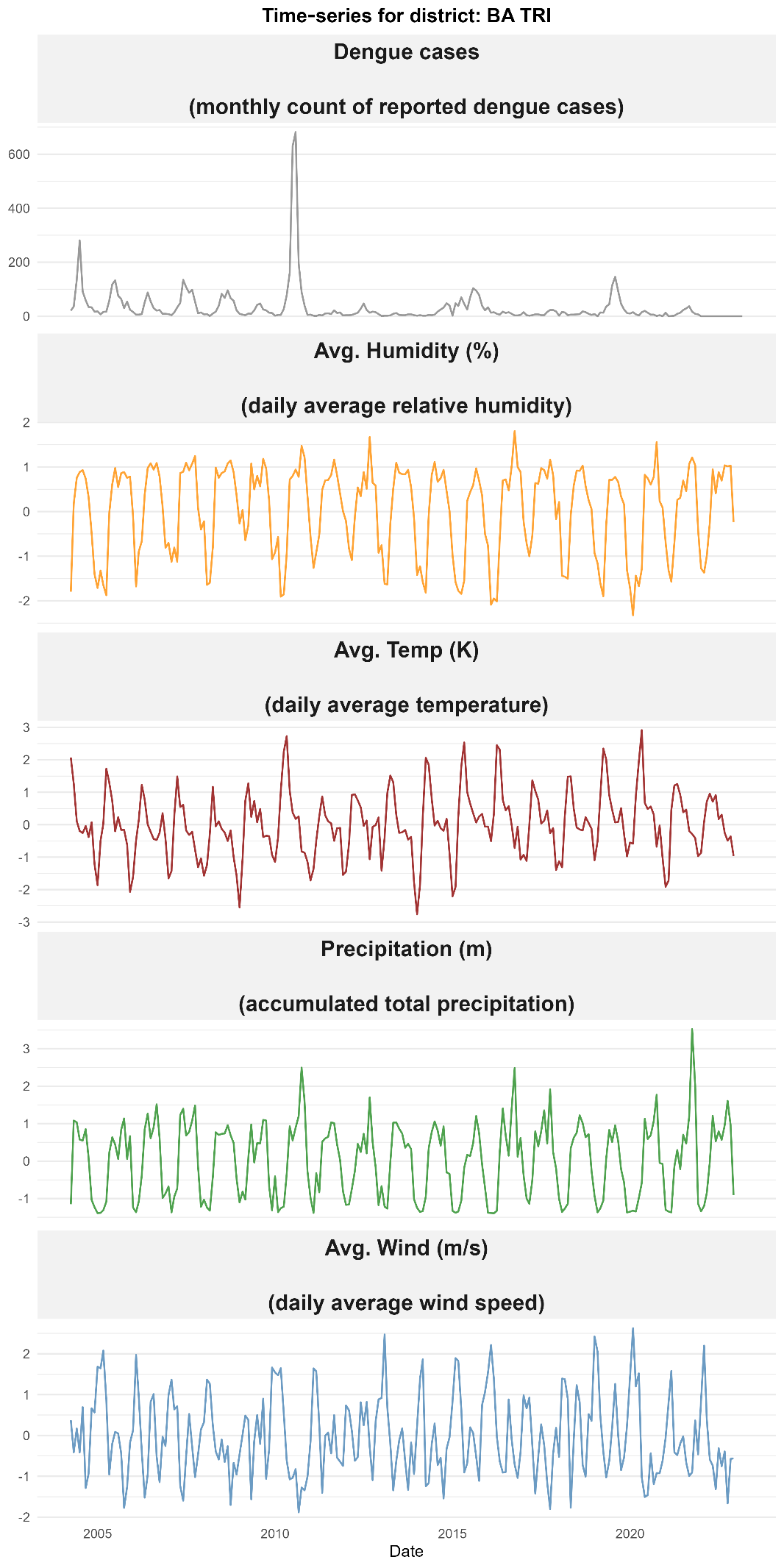

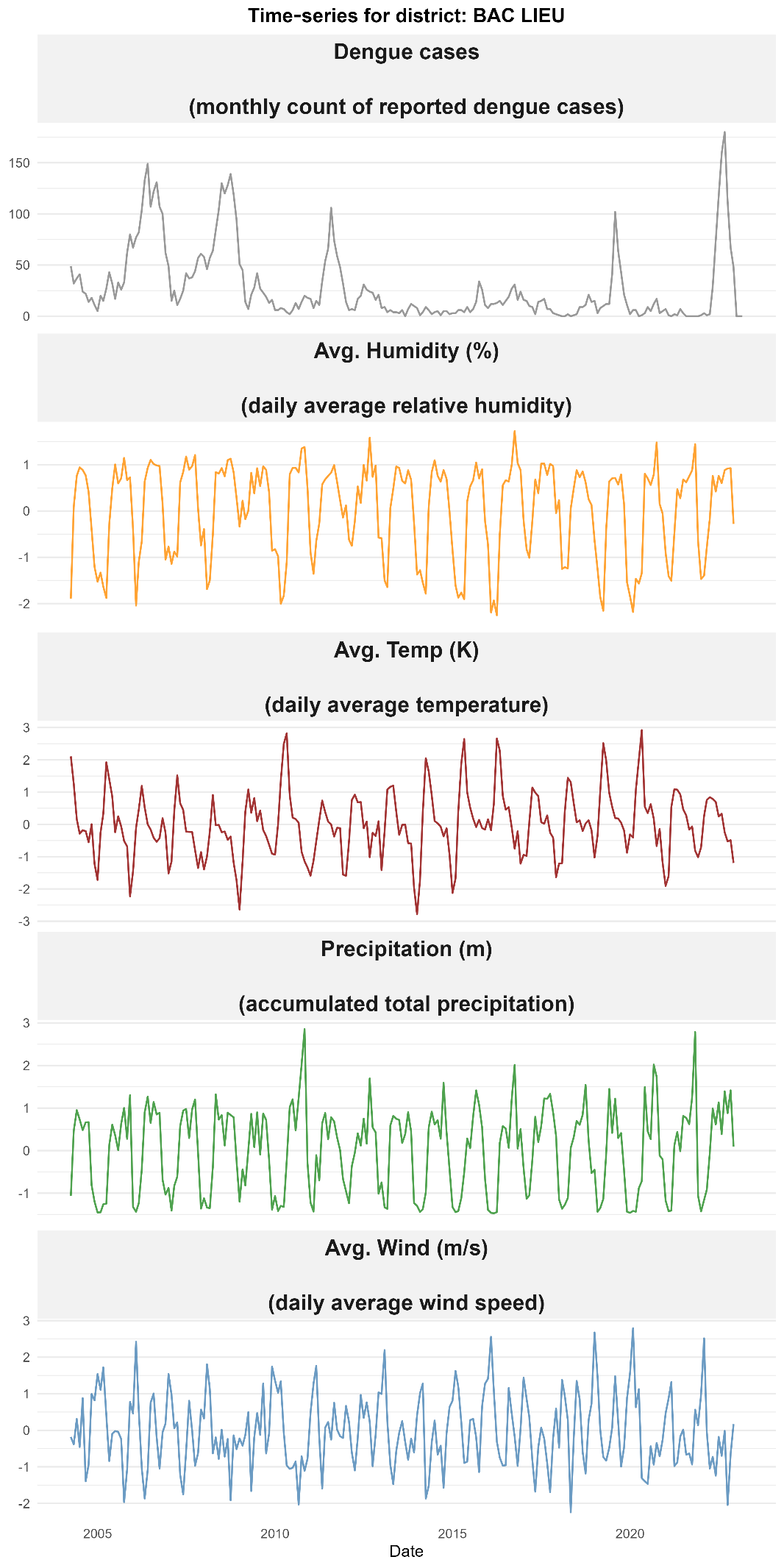

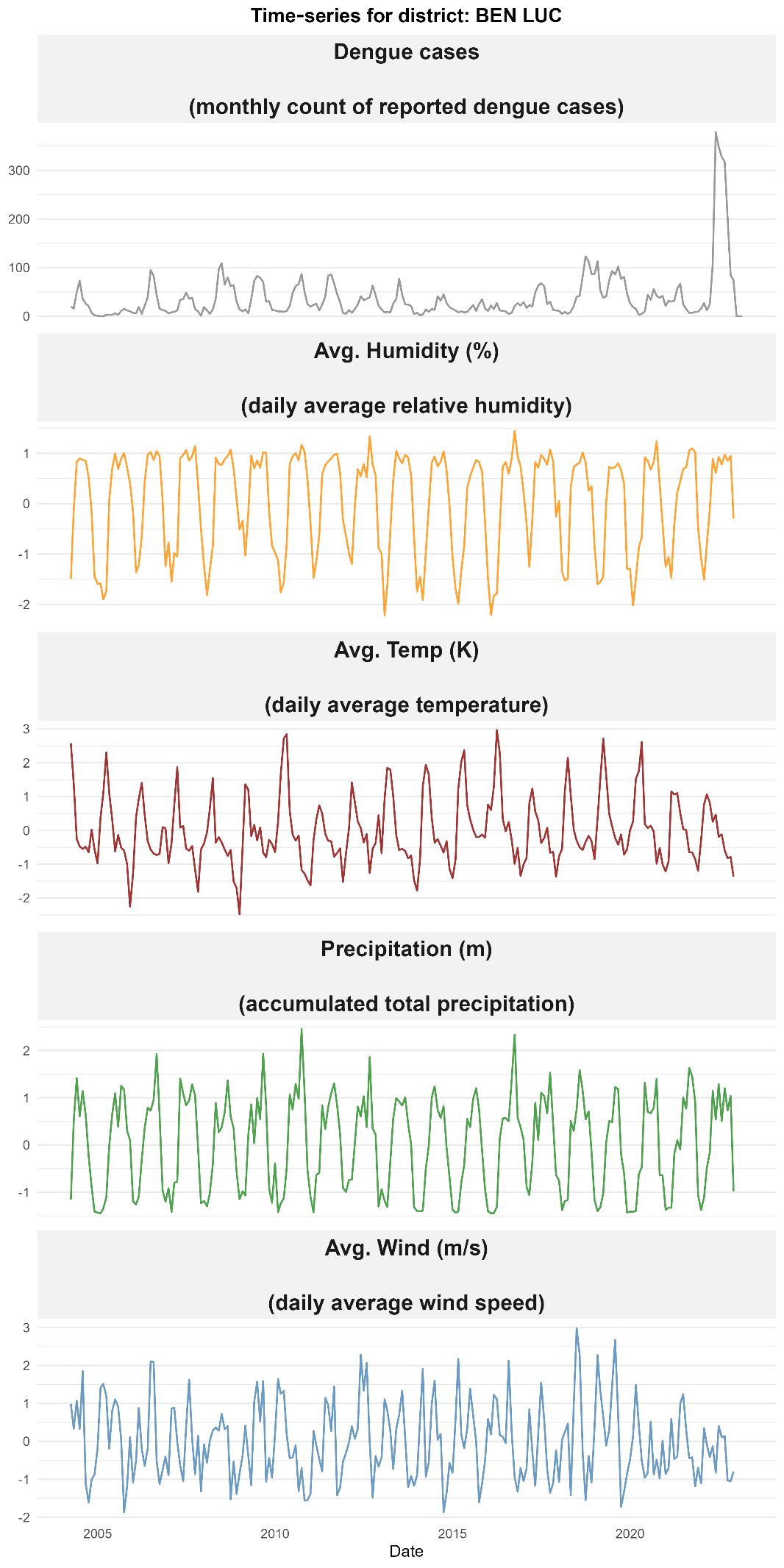

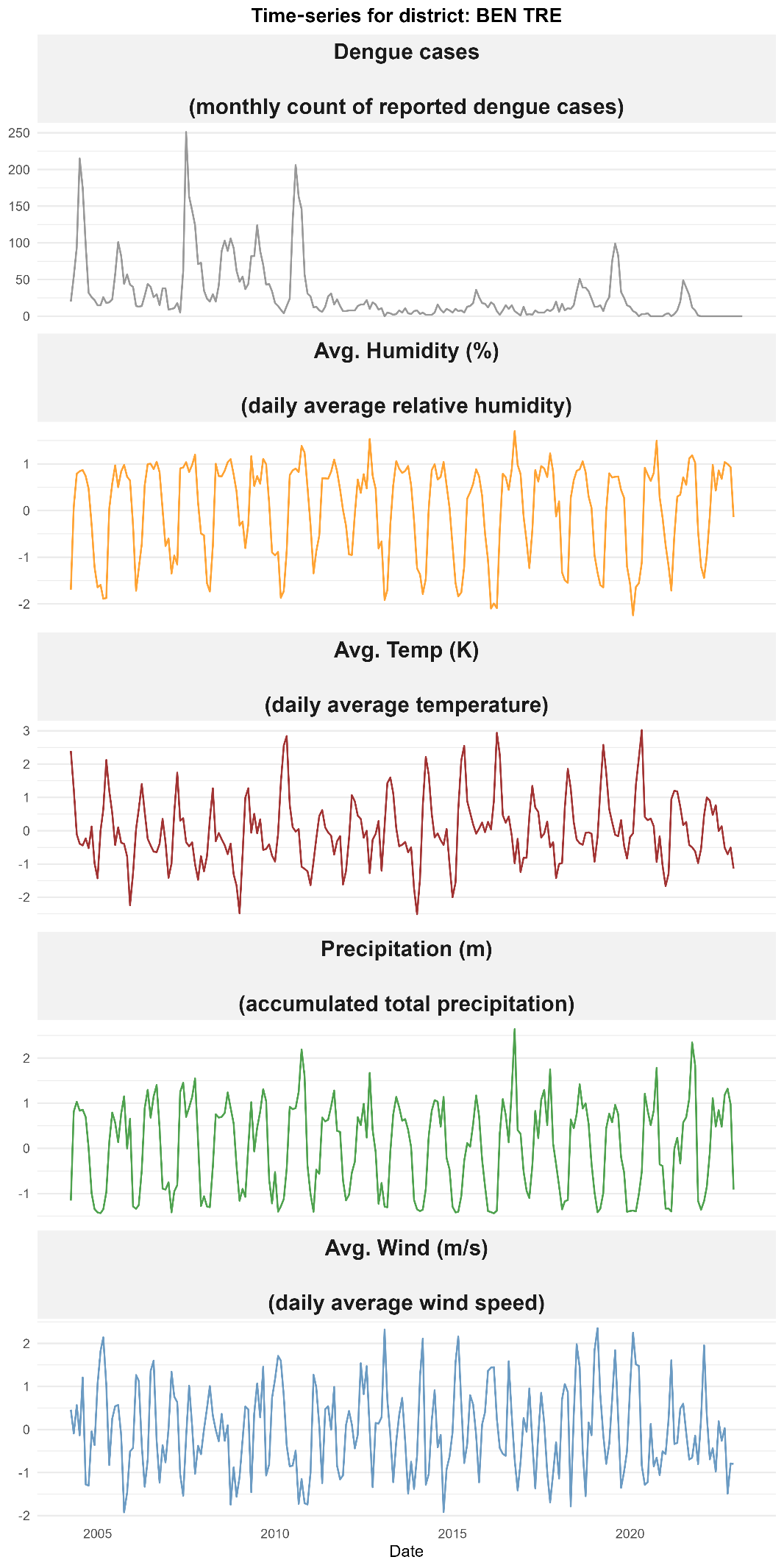

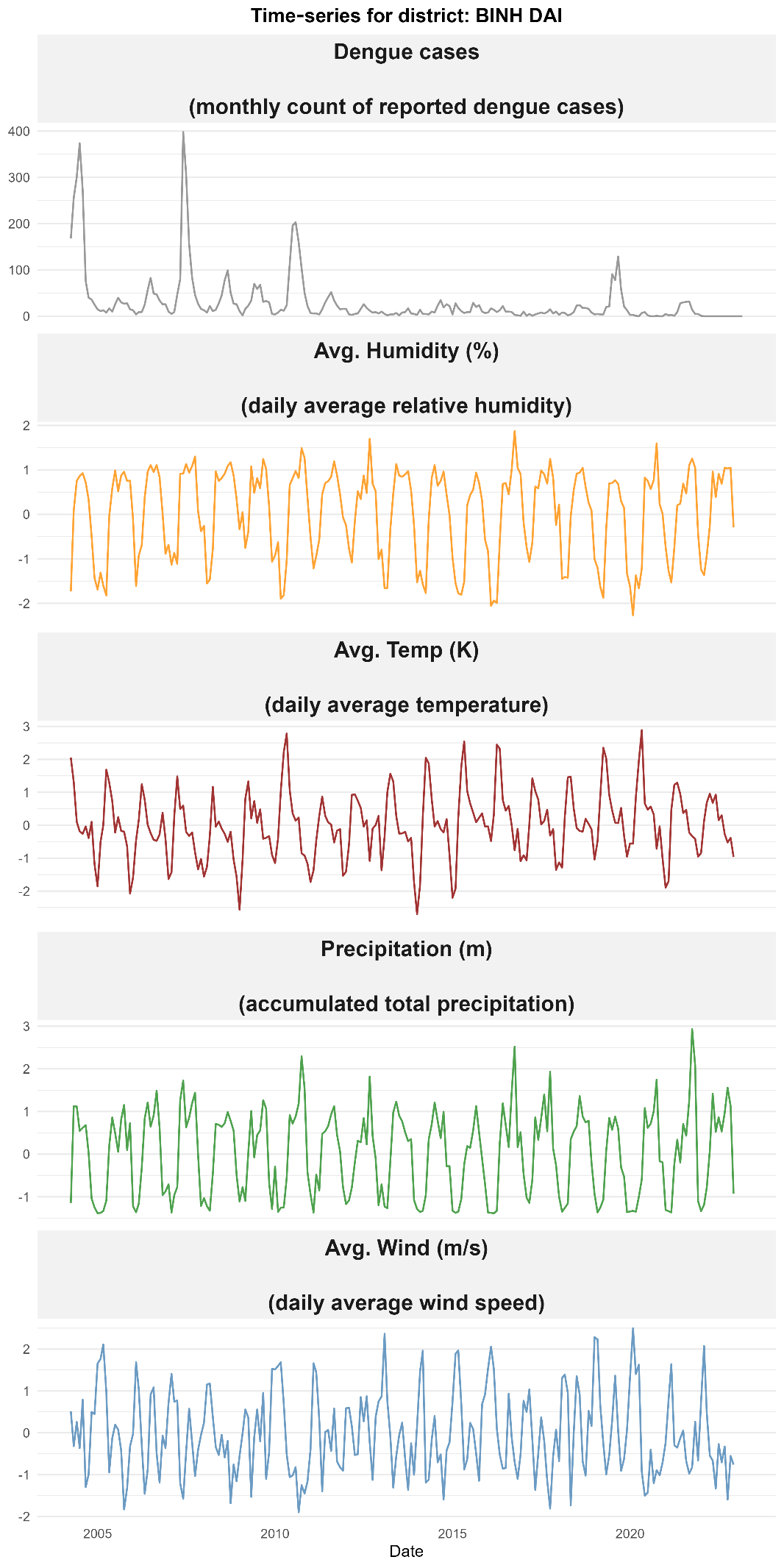

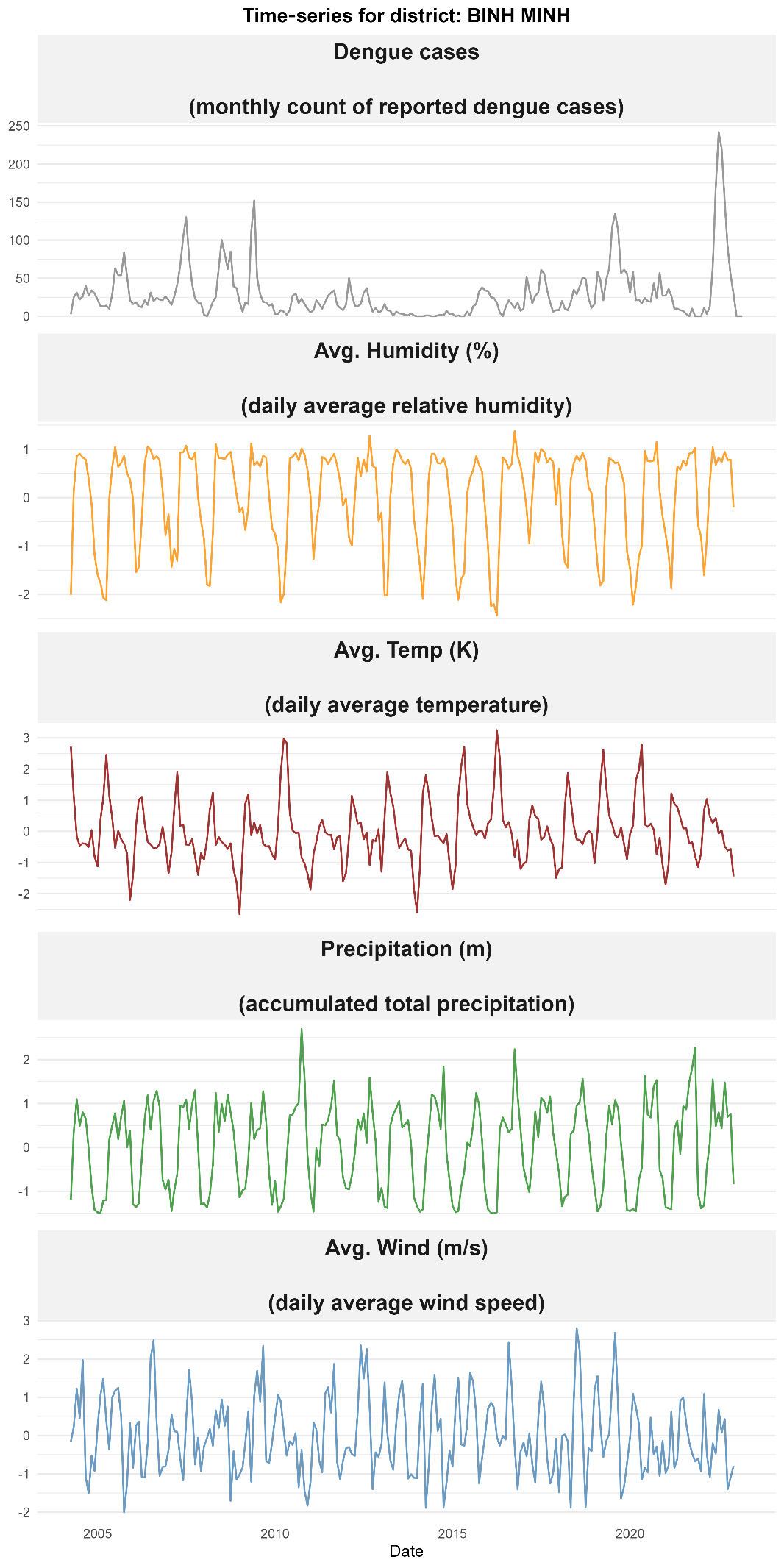

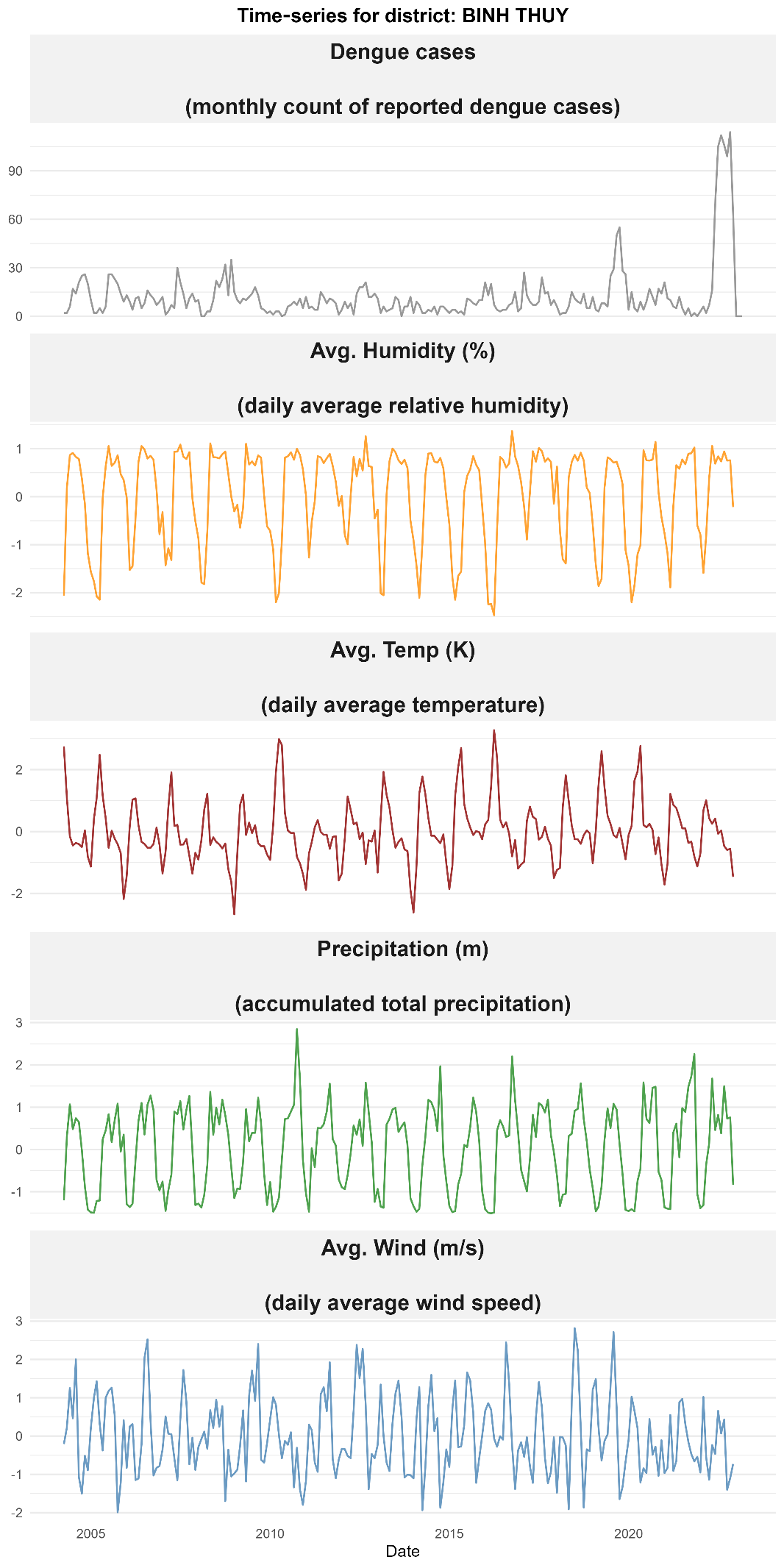

Supplement: S4 — (DOCX) [file pntd.0013571.s004.docx]
