# Supplementary material for: A district-level ensemble model to enhance dengue prediction and control for the Mekong Delta Region of Vietnam
Source: PLoS Negl Trop Dis. 2025 Sep 29;19(9):e0013571. doi: 10.1371/journal.pntd.0013571 (PMC12507206; doi:10.1371/journal.pntd.0013571)

**S5: Residuals vs. Fitted (Posterior Mean)**

All ensemble models exhibit errors clustered around zero, indicating that they are unbiased. At the low end, however, spatiotemporal 1 shows a wide “funnel” of significant negative errors, while spatiotemporal 2 and 3 keep errors much more even once predictions exceed about 50 cases. The hhh4 model is reliable for medium and high values but under-predicts very small counts (the striped pattern), suggesting its smoothing is overly strong on low values.

**
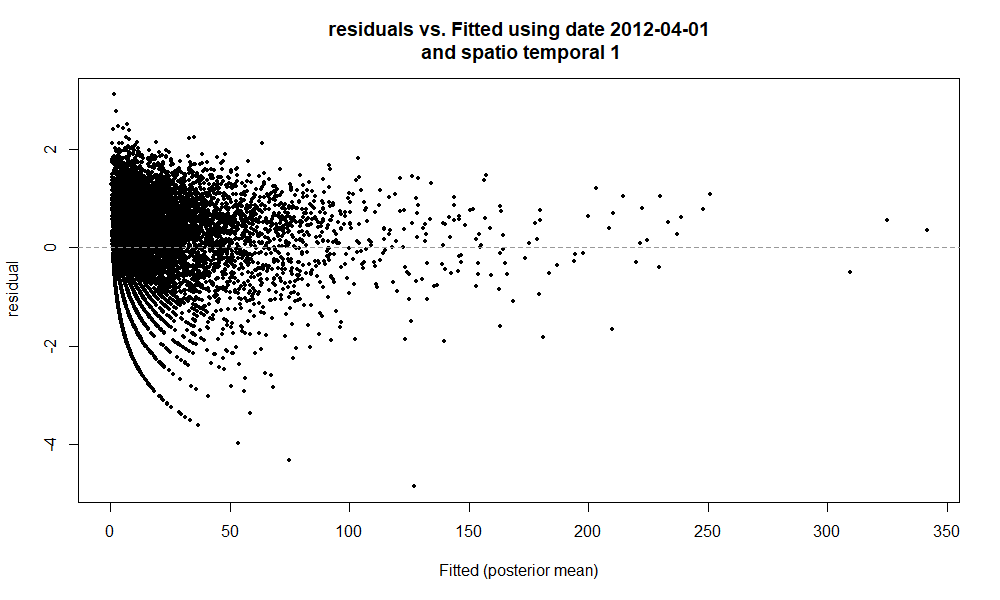
**
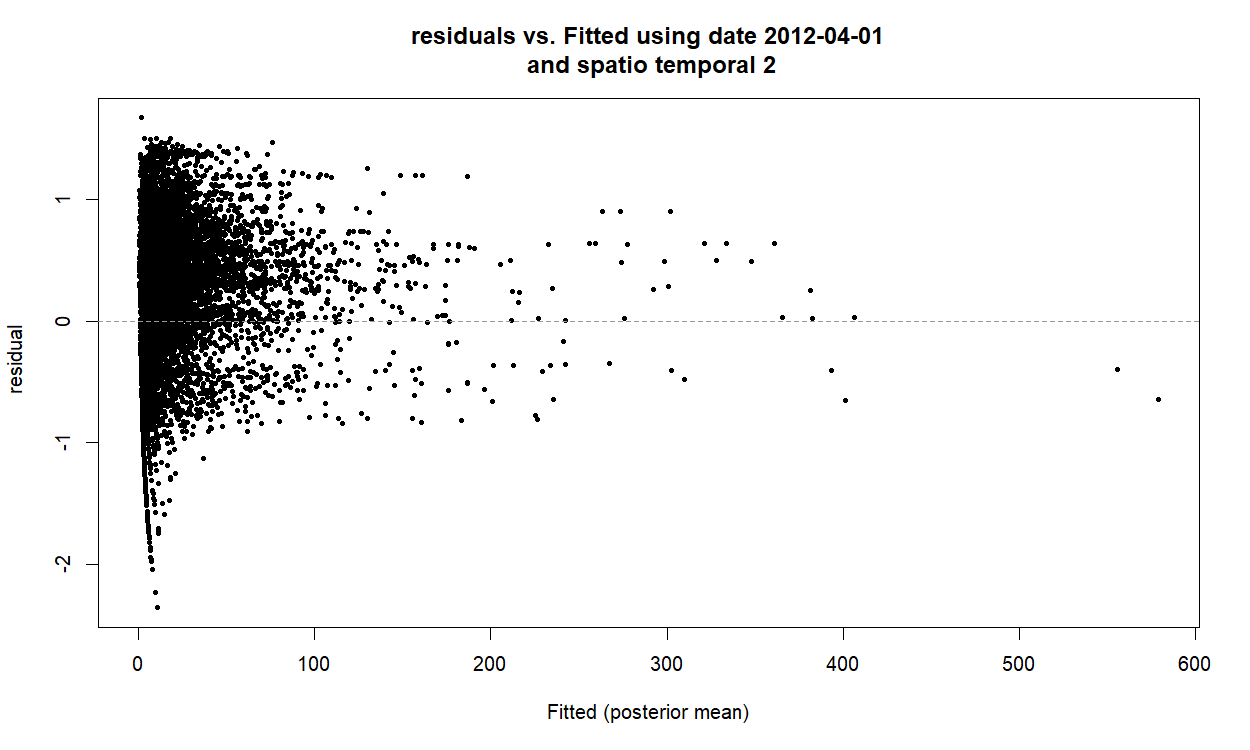


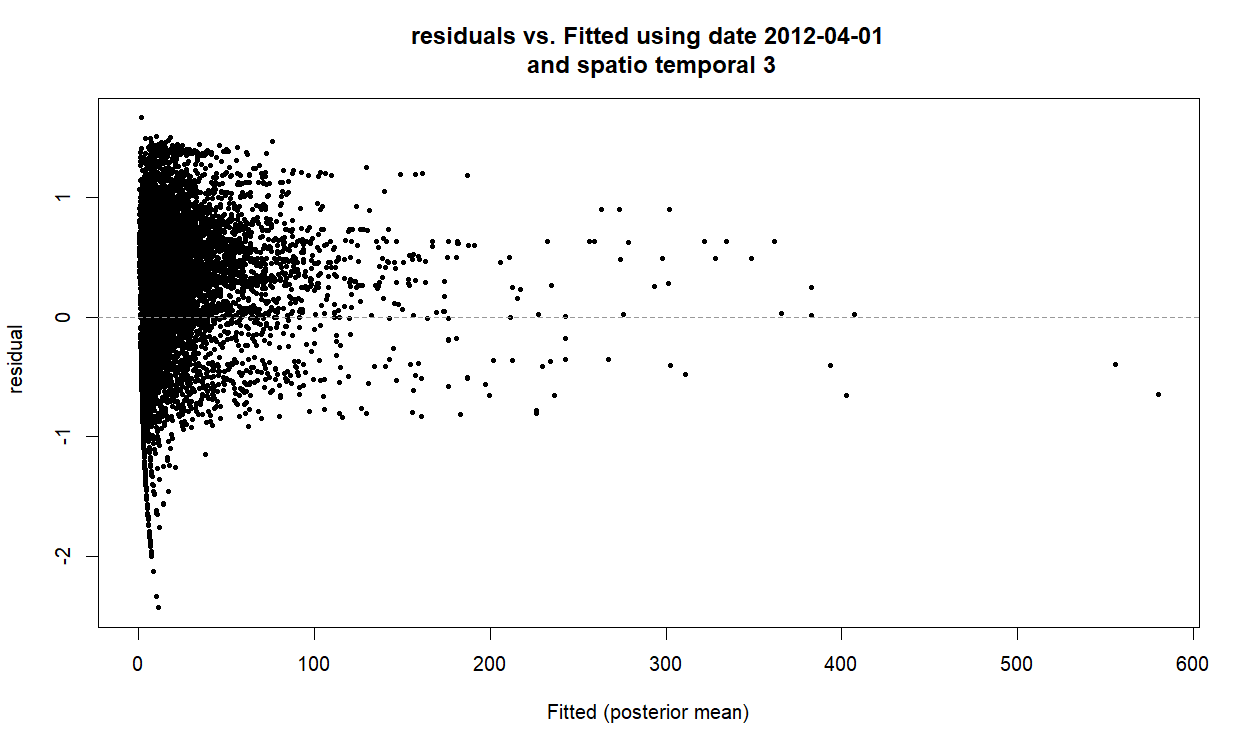

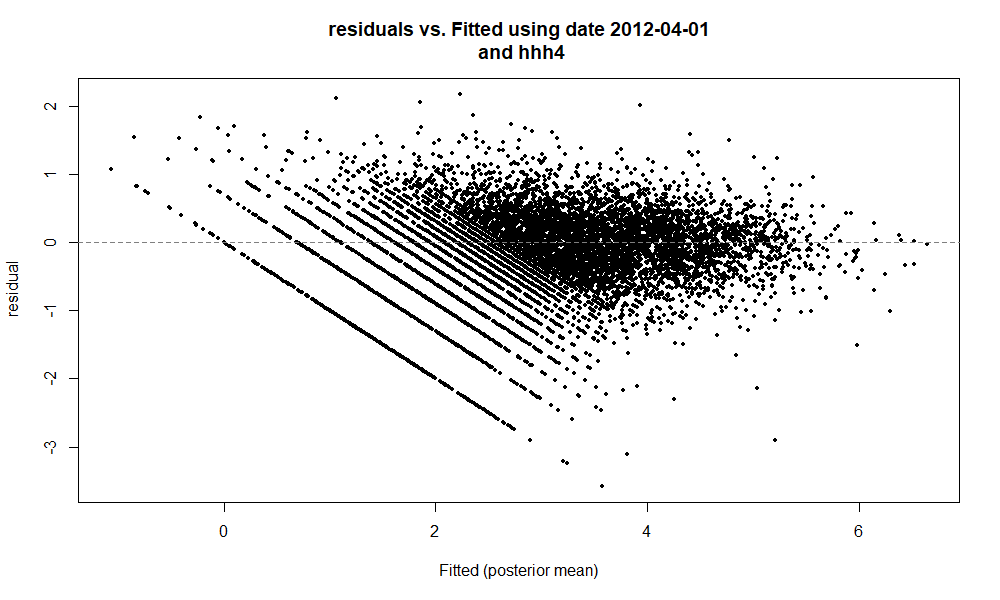

Supplement: S5 — (DOCX) [file pntd.0013571.s005.docx]
